# Characterization of circSEC11A as a novel regulator of Iodine-125 radioactive seed-induced anticancer effects in hepatocellular carcinoma via targeting ZHX2/GADD34 axis

**DOI:** 10.1038/s41420-023-01593-w

**Published:** 2023-08-10

**Authors:** Dong Li, Wujie Wang, Bin Liu, Die Jin, Yang Wang, Guanghui He, Lei Guo, Wen Liu, Yuliang Li

**Affiliations:** 1 Department of Interventional Medicine, The Second Hospital of Shandong University, Jinan, China; 2 Institute of Interventional Oncology, Shandong University, Jinan, China; 3 Department of Interventional Medicine, Weifang Second People’s Hospital, Weifang, China; 4 Department of Vascular Anomalies and Interventional Radiology, Children’s Hospital Affiliated to Shandong University, Jinan, China; 5 Fujian Provincial Key Laboratory of Innovative Drug Target Research, School of Pharmaceutical Sciences, Xiamen University, Xiamen, China

**Keywords:** Radiotherapy, Apoptosis

## Abstract

Iodine-125 (I-125) radioactive seed implantation is used for the local treatment of hepatocellular carcinoma (HCC), but the molecular mechanisms regulating its anticancer effects remain incompletely understood. In this study, we report that hsa_circ_0000647 (circSEC11A) is highly expressed after I-125 treatment in HCC cell lines and tissues and is a key regulator of I-125-induced anticancer effects. CircSEC11A acts as a competing endogenous RNA (ceRNA) to sponge miR-3529-3p, promoting the expression of zinc fingers and homeoboxes 2 (ZHX2) and enhancing I-125-induced anticancer effects. Dual-luciferase reporter assay, RNA pull-down, RNA immunoprecipitation, and fluorescence in situ hybridization were thereafter performed to verify the interaction among the molecules. Anticancer effects were detected using CCK-8, flow cytometry, TUNEL, EdU, transwell, and wound healing assays. Furthermore, ZHX2 transcriptionally inhibits GADD34, a negative regulator of endoplasmic reticulum stress (ERS), to enhance I-125- induced anticancer effects in vivo and in vitro. In conclusion, we characterized circSEC11A as a novel regulator of I-125-induced anticancer effects in HCC via miR-3529-3p/ZHX2/GADD34 axis-mediated ERS. Thus, circSEC11A may act as a potential therapeutic target for I-125 implantation in the clinic.

## Introduction

Hepatocellular carcinoma (HCC) is the second leading cause of cancer-related deaths in China [1]. Approximately 400,000 people die from liver cancer every year [2]. Approximately 55% of patients with HCC in China are at clinical stages III or IV at the time of diagnosis, and less than 30% can undergo surgical resection [3]. Under these conditions, interventional therapies, including transcatheter arterial chemoembolization (TACE), percutaneous ablation, and Iodine-125 (I-125) radioactive seed implantation, play an important role in the comprehensive treatment of HCC [4]. Unlike traditional external irradiation therapy, radioactive seed implantation is a low-dose-rate sustainable inter-tissue irradiation therapy with high conformability and sustained effectiveness [5]. Clinical studies have shown that I-125 seed implantation can increase the two-year survival rate of patients with HCC after TACE from 41 to 59% [6]. However, owing to the differences in the radiosensitivity of HCC tissues to I-125 among different patients, the treatment effect in patients with radioinsensitive HCC is not ideal. Therefore, the molecular mechanisms that regulate the induced anticancer effects of I-125 on HCC in clinical practice must be clarified.

Circular RNA (circRNA) was first discovered in the 1970s, and due to the technology of the time, it was thought to be a by-product of the splicing error of precursor RNAs with no functional potential [7]. However, circRNAs have been proven to be involved in the development of various cancers, including HCC, breast, lung, and pancreatic cancers [8]. CircRNAs can function through a variety of molecular mechanisms, the best known of which is to act as miRNA sponges [9]. Although studies have revealed the role of circRNAs in regulating HCC cell apoptosis and proliferation, few studies have explored the function of circRNAs in I-125-induced anticancer effects on HCC.

The zinc fingers and homeoboxes 2 (ZHX2) transcription factor, a member of the ZHX family, was first verified as an alpha-fetoprotein (AFP) repressor [10]. ZHX2 contains two zinc-finger domains and four homeodomains and acts as a transcription factor [11]. Although ZHX2 plays essential oncogenic roles in various cancers, its role in HCC is the opposite [12]. Studies have shown that ZHX2 acts as an oncogene in clear cell renal cell carcinoma and triple-negative breast cancer [13, 14]. However, ZHX2 has been verified as a tumor suppressor in HCC [15]. Furthermore, the functions of ZHX2 in cancer cell development, including apoptosis, metastasis, proliferation, and immunoregulation, have been identified [16–18]. Importantly, multidrug resistance mutation 1 (MDR1) is transcriptionally repressed by ZHX2 in HCC, revealing the role of ZHX2 in chemotherapy [11]. However, the relationship between ZHX2 expression and radiotherapy has not yet been clarified. Therefore, identifying the role of ZHX2 in regulating the I-125-induced apoptosis of liver cancer cells will further demonstrate the tumor-suppressive role of ZHX2 in HCC.

In this study, RNA-seq was performed to detect differentially expressed circRNAs and genes in I-125-trearted HCC cells and found that circ_000647 (circSEC11A), miR-3529-3p and ZHX2 were related to I-125-induced anticancer effects. The functions of circSEC11A/miR-3529-3p/ZHX2 axis in regulating the I-125-induced anticancer effects on HCC were preliminarily confirmed in vivo and in vitro. In addition, based on our previous study that suggested that the eIF2α-induced endoplasmic reticulum (ER) stress pathway regulates the I-125-induced anticancer effect on HCC, RNA- and chromatin immunoprecipitation (ChIP)-seq were performed. We found that the growth arrest and DNA damage-inducible protein (GADD34), a negative regulator of eIF2α phosphorylation, is a target of ZHX2. Taken together, the findings of the current study provide a theoretical basis for improving the clinical efficacy of I-125 seeds and discovering new therapeutic targets for HCC.

## Results

### Identification and characteristics of an I-125-related circRNA in HCC

**Fig. 1:**
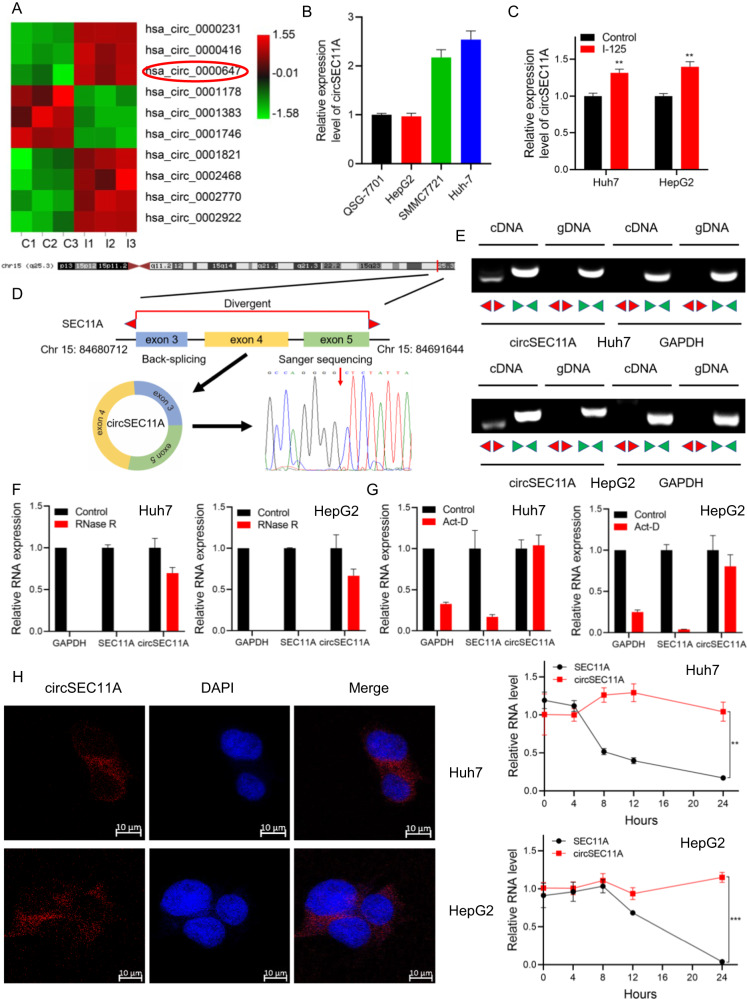
Identification and characteristics of an I-125-related circRNA in HCC. **A** Heatmap showing the up- and down-regulation circRNAs after treated with I-125 in HepG2 cells. **B** qRT-PCR for detecting the original expression level of circSEC11A in HepG2, QSG-7701, Huh7, and SMMC7721 cells. **C** qRT-PCR for detecting the expression level of circSEC11A in Huh7 and HepG2 cells. **D** Schematic diagram of chromosomal location and formation of circSEC11A. **E** Agarose gel electrophoresis was performed to detect the amplification of circSEC11A and GAPDH using convergent and divergent primers in cDNA and gDNA. **F** Relative RNA level of circSEC11A, linear SEC11A, and GAPDH treated with RNase R. **G** Relative RNA level of circSEC11A, linear SEC11A, and GAPDH treated with Act-D at the indicated time. **H** Flurorescence in situ hybridization (FISH) was used to detect the localization of circSEC11A (red) in Huh7 and HepG2 cells. Cell nuclei were stained with DAPI (blue). The data are presented as the mean ± SD. *P < 0.05, **P < 0.01, ***P < 0.001.

To identify potential circRNAs related to I-125 in HCC, circRNA-seq was performed on HepG2 cells. Among the circRNA-seq results, hsa_circ_0000647 was significantly different expressed in HepG2 cells in response to I-125 (Fig. 1A). More importantly, Kyoto Encyclopedia of Genes and Genomes (KEGG) pathway analysis of differential expressed circRNA showed that processing in the ER was enriched (Fig. S1). Furthermore, to determine which HCC cell lines to select for further experiments, the original expression of hsa _circ_0000647 was detected in HepG2, QSG7701, Huh7, and SMMC7721 cell lines. The results that showed that hsa_circ_0000647 was more highly expressed in Huh7 and SMMC7721 cells than in HepG2 and QSG-7701 cells; thus, the Huh7 and HepG2 cell lines were selected (Fig. 1B). To verify the circRNA-seq results, the expression of hsa_circ_0000647 was measured after treatment with I-125 (Fig. 1C). qPCR results showed that I-125 induced the upregulation of hsa_circ_0000647, which was consistent with the circRNA-seq results. The circBank and circBase databases showed that hsa_circ_0000647 was produced by back-splicing exons 3, 4, and 5 of the human SEC11A gene (Fig. 1D). Based on this, hsa _circ_0000647 was named circSEC11A. Sanger sequencing was performed to verify the circSEC11A sequence, and the junction site of circSEC11A was identified. Convergent and divergent primers were designed to clarify the characteristics of circSEC11A. Agarose gel electrophoresis showed that circSEC11A could be amplified from cDNA using both divergent and convergent primers. However, circSEC11A was only amplified from gDNA using convergent primers, and GAPDH was amplified from cDNA and gDNA using only convergent primers (Fig. 1E). The stability of circRNAs is stronger than that of linear RNA, and RNase R and actinomycin D (Act-D) were used to verify this characteristic. RNA extracted from HepG2 or Huh7 cells was treated with RNase R, and qRT-PCR results suggested that compared to SEC11A or GAPDH, circSEC11A was significantly more resistant to RNase R (Fig. 1F). In addition, compared to SEC11A and GAPDH, circSEC11A was more stable against Act-D in a time-dependent manner (Fig. 1G). To investigate the location of circSEC11A, fluorescence in situ hybridization (FISH) was performed to detect whether circSEC11A was in the nucleus or cytoplasm. The results showed that the majority of circSEC11A was in the cytoplasm of HepG2 and Huh7 cells. Thus, we identified circSEC11A as a potential circRNA related to the I-125-induced anticancer effects on HCC. Its sequence and biological characteristics were verified (as circRNA) and its location was mainly in the cytoplasm.

### CircSEC11A promotes the I-125-induced anticancer effect on HCC

**Fig. 2:**
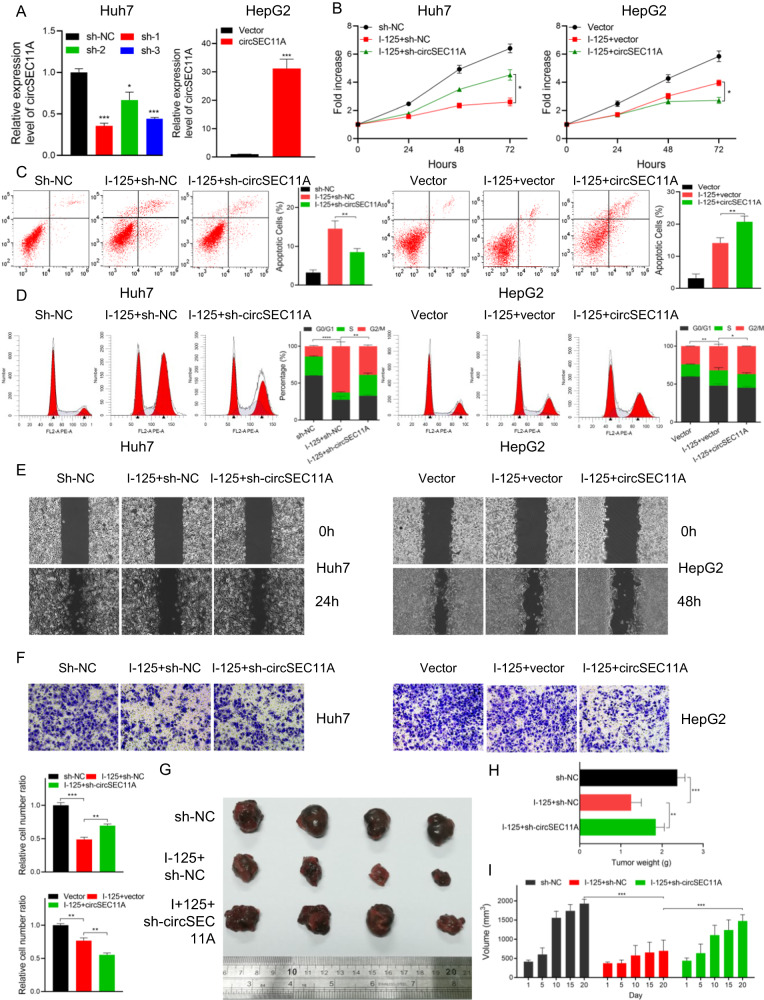
CircSEC11A promotes the I-125-induced anticancer effect on HCC. **A** The regulatory effect verification of sh-NC/sh-circSEC11A and Vector/circSEC11A in Huh7 and HepG2 cells. **B**, **C** CCK-8 assay and flow cytometry for cell cycle were performed to detect cell proliferation ability in Huh7 and HepG2 cells transfected with sh-circSEC11A or circSEC11A and treated with I-125. **D**–**F** Flow cytometry, wound healing and transwell assay for detecting cell apoptosis and cell migration ability in Huh7 and HepG2 cells transfected with sh-circSEC11A or circSEC11A and treated with I-125. **G**–**I** Using in vivo I-125 irradiation model, tumor volume and tumor weight were measured. The data are presented as the mean ± SD. *P < 0.05, **P < 0.01, ***P < 0.001.

To explore the role of circSEC11A in the I-125-induced anticancer effects on HCC, based on the original expression levels of circSEC11A, circSEC11A was knocked down in Huh7 cells, which normally express higher circSEC11A levels, and overexpressed in HepG2 cells, which normally express lower circSEC11A levels (Fig. 2A). The regulatory effect was verified using qRT-PCR, and sh-1 showed the most significant knockdown effect. To detect cell proliferation, a CCK-8 assay and flow cytometry were performed. As shown in Fig. 2B, although I-125 significantly inhibited cell growth, circSEC11A knockdown reversed this effect in Huh7 cells. In contrast, circSEC11A overexpression promoted the I-125-induced inhibition of cell growth in HepG2 cells. In addition, I-125 increased the G2/M phase ratio from 13.86 ± 0.54 to 62.83 ± 3.3, which could be attenuated to 38.86 ± 1.03 by downregulating circSEC11A in Huh7 cells (Fig. 2C). However, overexpression of circSEC11A increased the I-125-induced G2/M phase cell cycle arrest rate from 31.01 ± 1.5 to 37.05 ± 0.06 in HepG2 cells. Flow cytometric analysis revealed that I-125-induced apoptosis was compromised after circSEC11A knockdown, whereas it was significantly promoted after circSEC11A overexpression (Fig. 2D). To further investigate whether circSEC11A regulates the I-125-induced anti-migration effect in HCC, wound healing and transwell assays were performed. As shown in Fig. 2E, although I-125 significantly reduced the wound closure rate, circSEC11A downregulation attenuated this effect in Huh7 cells, and circSEC11A upregulation boosted this effect in HepG2 cells. The results are shown in Fig. S2. The transwell assay demonstrated similar results (Fig. 2F). Furthermore, the role of circSEC11A in I-125-induced anticancer effects was verified in vivo. Consistent with the results of the in vitro experiments, I-125 significantly inhibited the tumor growth rate, compared to the control group, and tumor volume was decreased from 1931 ± 55.58 mm^3^ to 696.7 ± 140.5 mm^3^. Downregulating circSEC11A reversed the I-125-induced inhibition of tumor growth and the tumor volume was increased to 1477 ± 81.69 mm^3^ (Fig. 2G–I). Moreover, the tumor weight in the I-125 group was reduced from 1.84 ± 0.10 g to 1.25 ± 0.11 g. Thus, circSEC11A promotes the induced anticancer effects of I-125 on HCC cells.

### CircSEC11A promotes the I-125-induced anticancer effect in HCC via functions as a miR-3529-3p sponge

**Fig. 3:**
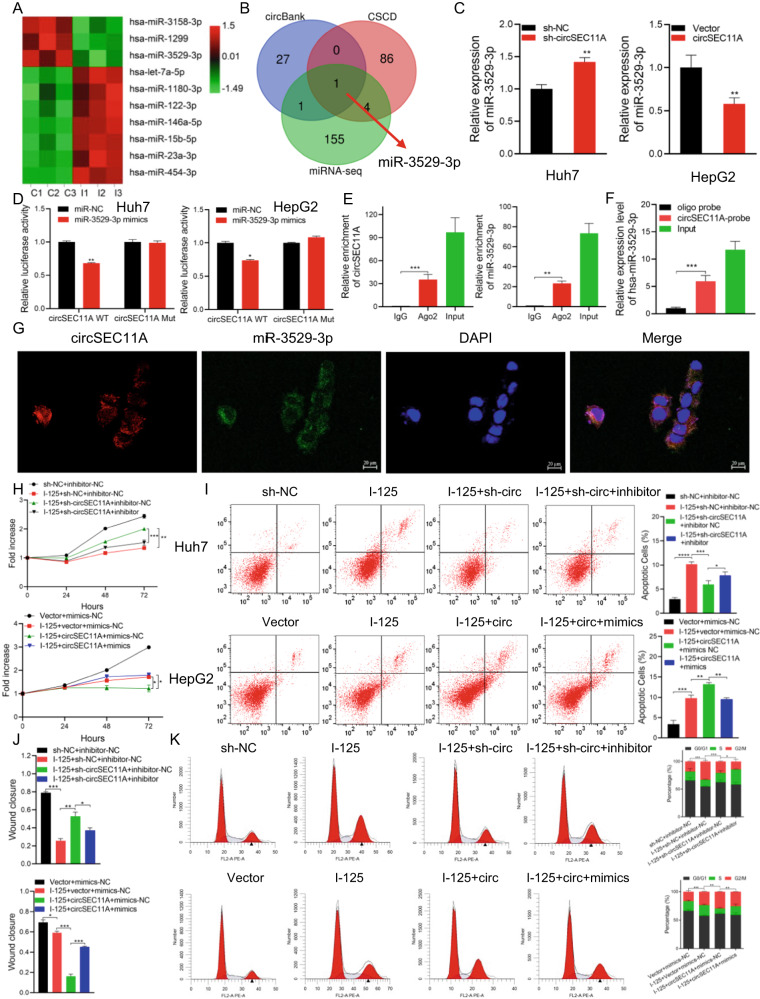
CircSEC11A promotes the I-125-induced anticancer effect in HCC via functions as a miR-3529-3p sponge. **A** The heatmap of the differentially expressed miRNAs in HepG2 cells treated with I-125. **B** Veen diagram to identify miRNA predicted to bind with circSEC11A by circBANK (www.circbank.cn), CSCD (gb.whu.edu.cn/CSCD), and miRNA-seq (Control vs I-125). **C** qRT-PCR to detect the expression level of miR-3529-3p after circSCE11A was down- or up-regulated in Huh7 or HepG2 cells. **D** Relative luciferase activity were detected in cells co-transfected with circSEC11A-WT/circSEC11A-Mut and miR-NC/miR-3529-3p mimics in Huh7 and HepG2 cells. **E** RIP was performed with anti-AGO2 or IgG antibodies and the enrichment of circSEC11A and miR-3529-3p were detected via qRT-PCR in Huh7 cells. **F** RNA pull-down assay was performed using a circSEC11A probe and the enrichment of miR-3529-3p was detected via qRT-PCR in Huh7 cells. **G** The location of circSEC11A and miR-3529-3p in Huh7 cells were detected with FISH assay. **H**–**K** CCK-8, flow cytometry, and wound healing assay were performed to detect cell proliferation, apoptosis, and metastasis in HepG2 and Huh7 cells treated with I-125 and co-transfected with sh-NC/sh-circSEC11A and miR-3529-3p NC/inhibitor or vector/circSEC11A and miR-3529-3p NC/mimics. The data are presented as the mean ± SD. *P < 0.05, **P < 0.01, ***P < 0.001.

Based on the location of circSEC11A—mainly found in the cytoplasm—competing endogenous RNA (ceRNA) is the most reported mechanism by which circRNAs act as miRNA sponges. To define the miRNAs, circBANK and CSCD databases were used. More importantly, miRNA-seq was performed on HepG2 cells treated with I-125 (Fig. 3A). After crossing these predicted and screened miRNAs, only miR-3529-3p was selected (Fig. 3B). Next, the expression level of miR-3529-3p was detected in Huh7 and HepG2 cells transfected with sh-NC/sh-circSEC11A or vector/circSEC11A, and the results showed that circSEC11A negatively regulated miR-3529-3p (Fig. 3C). Furthermore, two luciferase reporter plasmids with the wild-type and miR-3529-3p binding site mutant were constructed (Fig. S3A). The luciferase activity of the wild-type miR-3529-3p was significantly inhibited by miR-3529-3p mimics in Huh7 and HepG2 cells, whereas the mutant showed no obvious change, indicating that miR-3529-3p could not only bind to circSEC11A but also negatively regulate its expression (Fig. 3D). The RNA immunoprecipitation (RIP) assay showed that both circSEC11A and miR-3529-3p could directly bind to Ago2 in Huh7 cells (Fig. 3E). A circSEC11A probe was constructed and used to perform an RNA pull-down assay in Huh7 cells. qRT-PCR results revealed that miR-3529-3p was significantly enriched (Fig. 3F). In addition, the results of the FISH assay suggested that circSEC11A and miR-3529-3p colocalized in the cytoplasm of Huh7 cells (Fig. 3G). After verifying the interaction between circSEC11A and miR-3529-3p, we determined whether miR-3529-3p could reverse the function of circSEC11A in the I-125-induced anticancer effects on HCC. The CCK-8 results demonstrated that the downregulation of circSEC11A could reverse the I-125-induced antiproliferative effect on Huh7 cells, which could be attenuated by inhibiting miR-3529-3p. Overexpression of miR-3529-3p enhanced the I-125-induced antiproliferative effect on HepG2 cells, which was reversed by the overexpression of miR-3529-3p (Fig. 3H). Similar results were obtained using flow cytometry and wound healing assays (Fig. 3I–K and Fig. S3B).

### CircSEC11A/miR-3529-3p/ZHX2 axis promotes I-125-induced anticancer effects in HCC

**Fig. 4:**
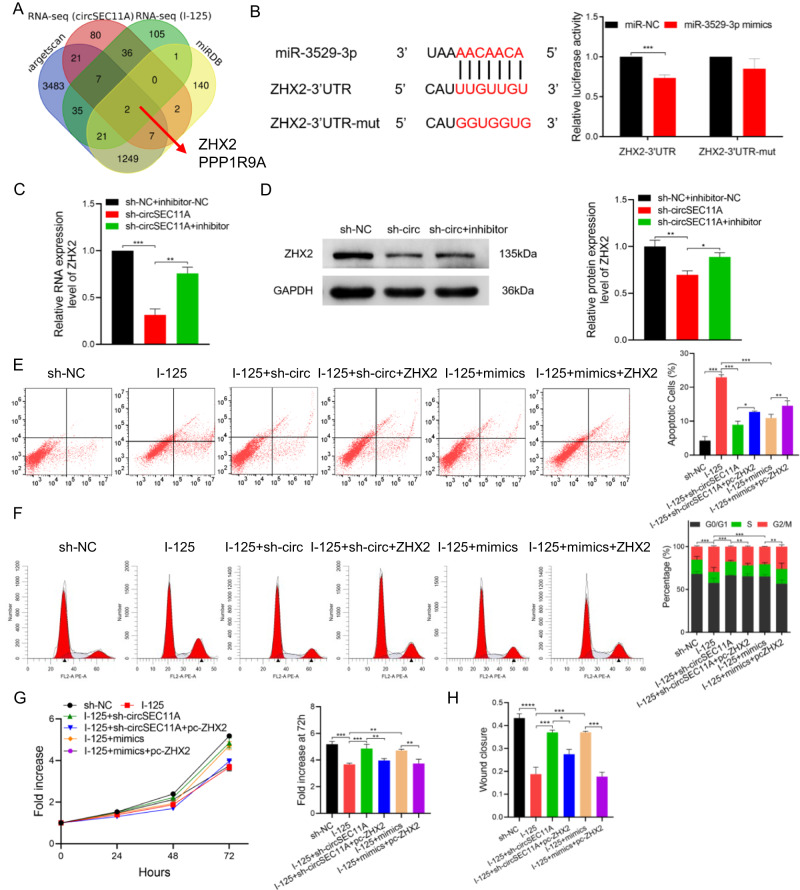
CircSEC11A/miR-3529-3p/ZHX2 axis promotes I-125-induced anticancer effects in HCC. **A** Veen diagram to identify mRNA predicted to bind with miR-3529-3p by TargetScan (www.targetscan.org), miRDB (mirdb.org), RNA-seq (Control vs I-125), and RNA-seq (NC vs sh-circSEC11A). **B** Relative luciferase activity was detected in Huh7 cells co-transfected with miR-NC/miR-3529-3p mimics and ZHX2-3’UTR/ZHX2-3’UTR-mut. **C**, **D** The expression of ZHX2 in RNA and protein level were detected in Huh7 cells co-transfected with sh-NC/sh-circSEC11A and miR-NC/miR-3529-3p inhibitor. **E**–**H** Flow cytometry, CCK-8, and wound healing assay were performed to detect cell proliferation, apoptosis, and metastasis in Huh7 cells treated with I-125 and co-transfected with sh-NC/sh-circSEC11A, miR-NC/miR-3529-3p mimics, or pcDNA/pcZHX2. The data are presented as the mean ± SD. *P < 0.05, **P < 0.01, ***P < 0.001.

To identify the potential targets of miR-3529-3p, RNA-seq was performed using HepG2 cells treated with I-125 or transfected with sh-circSEC11A. Moreover, to find the targets more accurately, TargetScan and miRDB database were used. Combined with high-throughput sequencing data and bioinformatic results, ZHX2 and PPP1R9A were selected (Fig. 4A). Based on the published studies suggesting that the relationship between ZHX2 and the occurrence and development of HCC is clearer, ZHX2 was identified as target gene to study [15]. Next, to investigate whether miR-3529-3p could bind to the 3’UTR of ZHX2, luciferase reporters of wild-type ZHX2 (ZHX2-3’UTR-WT) and mutant ZHX2 (ZHX2-3’UTR-Mut) were constructed (Fig. 4B). The results of the dual-luciferase report assay in Huh7 cells showed that the luciferase activity of ZHX2-3’UTR-WT was decreased by miR-3529-3p mimics, whereas that of the ZHX2-3’UTR-Mut showed no significant difference, suggesting that miR-3529-3p could not only bind to the 3’UTR of ZHX2, but also negatively regulate the ZHX2 expression level (Fig. 4B). Then, whether ZHX2 could be regulated by circSEC11A/miR-3529-3p was verified using qRT-PCR and western blot analyses by detecting the expression level of ZHX2 after transfections with sh-circSEC11A and the miR-3529-3p inhibitor. The results demonstrated that downregulation of circSEC11A reduced the expression of ZHX2, whereas the inhibition of miR-3529-3p reversed this effect in Huh7 cells (Fig. 4C, D). However, whether the I-125-induced anticancer effect can be regulated via the circSEC11A/miR-3529-3p/ZHX2 axis via cell proliferation, apoptosis, and metastasis remains unclear. As shown in Fig. 4E, the I-125-induced antiproliferative effect could be reversed via downregulation of circSEC11A with sh-circSEC11A, which could be attenuated by the overexpression of ZHX2 with pcZHX2. Moreover, miR-3529-3p mimics could reduce the I-125-indcued anti-proliferation effect, which was reversed by the overexpression of ZHX2 with pcZHX2. Regarding cell apoptosis and metastasis, the results of flow cytometry and wound healing assays were similar to those of the cell proliferation assays (Fig. 4F–H and Fig. S4). Thus, the circSEC11A/miR-3529-3p/ZHX2 axis regulates the anticancer effects that I-125 exerts on HCC.

### ZHX2 is an essential gene of I-125-induced anticancer effect in HCC

**Fig. 5:**
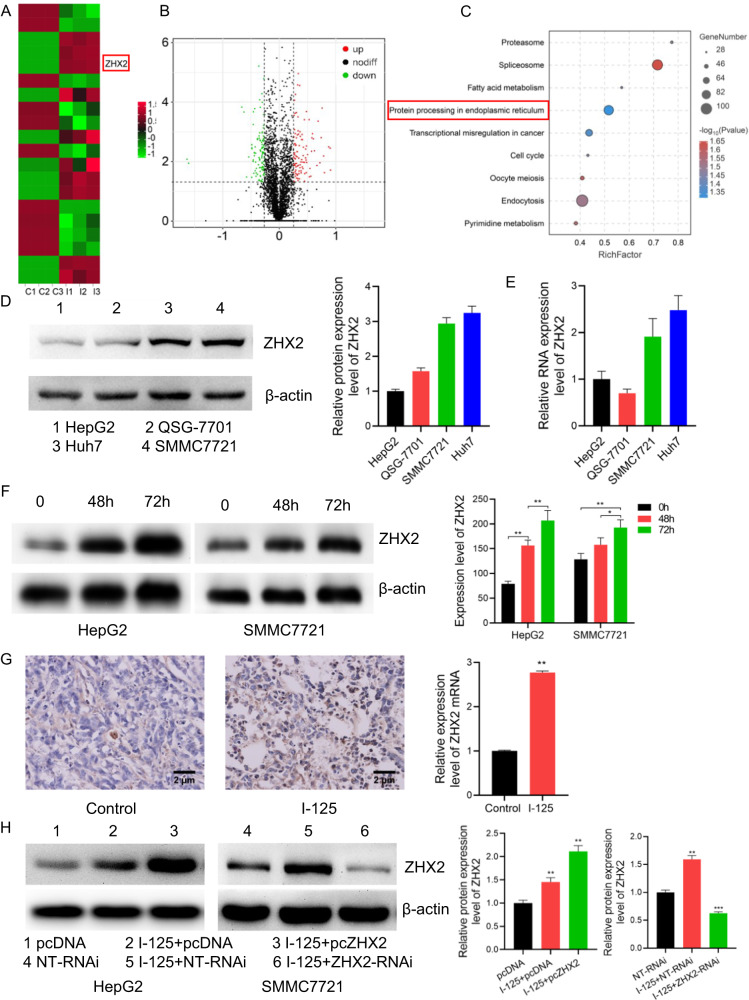
ZHX2 is an essential gene of I-125-induced anticancer effect in HCC. **A**, **B** The heatmap and volcano map of the differentially expressed mRNAs identified via RNA-seq using HepG2 cell treated with or without I-125. **C** The KEGG pathway enrichment of the differentially expressed mRNA. **D**, **E** The western blot and qRT-PCR for detecting ZHX2 expression level in HepG2, QSG-7701, Huh7, and SMMC7721 cells. **F**, **G** The western blot and IHC for detecting ZHX2 expression level in vitro and in vivo after treated with I-125. **H** The verification of transfection of pcDNA/pcZHX2 or NT-RNAi/ZHX2-RNAi in HepG2 and SMMC7721 cells. The data are presented as the mean ± SD. *P < 0.05, **P < 0.01.

The RNA-seq results, performed with HepG2 cells treated with I-125, revealed that 121 genes were upregulated and 86 were downregulated (fold change ≥ |1.2| and *P* < 0.05). Among the differentially expressed genes, ZHX2 was significantly upregulated after I-125 treatment (Fig. 5A, B). After clarifying the role of ZHX2 in circSEC11A/miR-3529-3p axis regulating I-125-induced anticancer effect, the role of ZHX2 as a transcription factor in regulating the function of I-125 is worthy investigation. KEGG cluster analysis revealed several enriched pathways, including protein processes in the ER, transcriptional misregulation in cancer, and the cell cycle (Fig. 5C). To better understand the role of ZHX2 in HCC, several common HCC cell lines, including HepG2, QSG-7701, Huh7, and SMMC7721, were used to determine the expression levels of ZHX2. The results showed that the protein and RNA expression levels of ZHX2 were relatively low in HepG2 and QSG-7701 cells and high in Huh7 and SMMC7721 cells (Fig. 5D, E). Based on these results, HepG2 and SMMC7721 cells were selected for further experiments. Using in vitro and in vivo I-125 radiation models, we found that ZHX2 was upregulated after treatment with I-125 (Fig. 5F, G). Furthermore, pcDNA or pcZHX2 was transfected into HepG2 cells to overexpress ZHX2, and NT-RNAi or ZHX2-RNAi was transfected into SMMC7721 cells to knockdown ZHX2. The transfection effects were verified (Fig. 5H). Taken together, our findings revealed that ZHX2 was upregulated after treatment with I-125 and that it is a potential marker for regulating the I-125-induced anticancer effects on HCC.

### ZHX2 enhances the I-125-induced anticancer effect in HCC

**Fig. 6:**
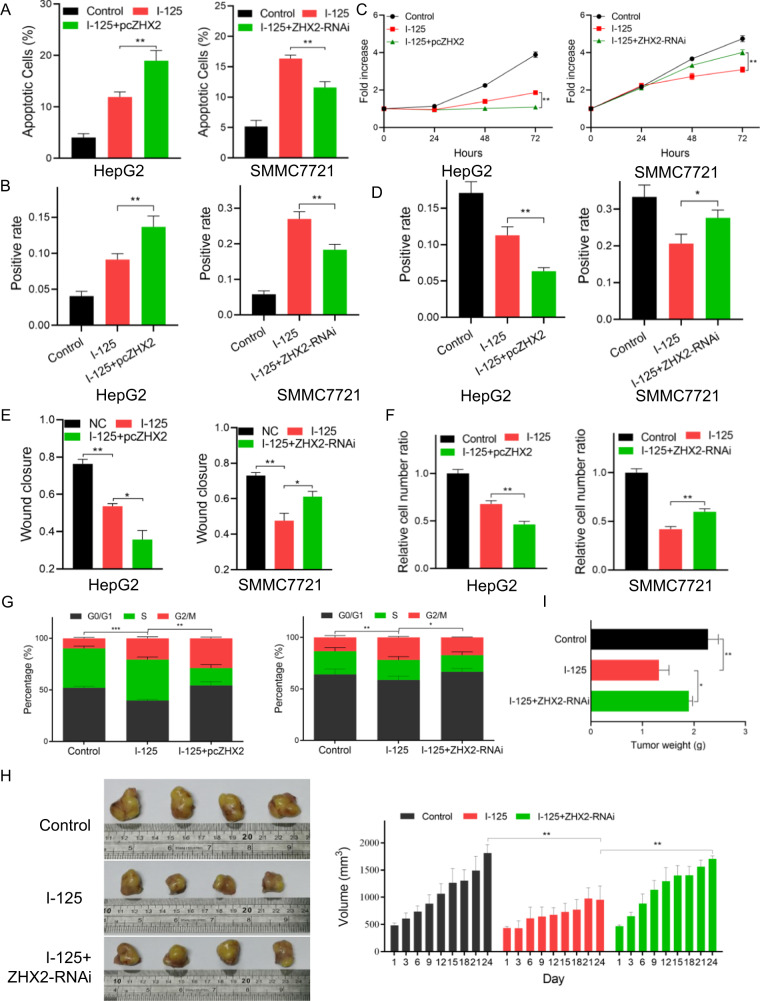
ZHX2 enhances the I-125-induced anticancer effect in HCC. **A**, **B** Flow cytometry and TUNEL staining were performed to detect the cell apoptosis in HepG2 and SMMC7721 cells treated with I-125 and transfected with pcZHX2 or ZHX2-RNAi. **C**, **D** CCK-8 assay and EdU staining were performed to detect the cell proliferation in HepG2 and SMMC7721 cells treated with I-125 and transfected with pcZHX2 or ZHX2-RNAi. **E**, **F** Wound healing and transwell assay were used to verify the cell migration ability in HepG2 and SMMC7721 cells treated with I-125 and transfected with pcZHX2 or ZHX2-RNAi. **G** Flow cytometry was performed to detect the cell cycle distribution in HepG2 and SMMC7721 cells treated with I-125 and transfected with pcZHX2 or ZHX2-RNAi. **H**, **I** Using in vivo I-125 irradiation model, tumor volume and weight were measured after treated with I-125 or transfected with ZHX2-RNAi. The data are presented as the mean ± SD. *P < 0.05, **P < 0.01, ***P < 0.001.

To explore the role of ZHX2 in the I-125-induced anticancer effects on HCC, ZHX2 was overexpressed or knocked down in HepG2 and SMMC7721 cells, as mentioned above, and the cells were then treated with I-125. For apoptotic analysis, flow cytometry and TUNEL staining were performed. Overexpression of ZHX2 increased the I-125-induced apoptosis rate in HepG2 cells from 11.91 ± 0.56 to 18.96 ± 1.15 (Fig. 6A). On the contrary, ZHX2 knockdown significantly decreased the I-125-induced apoptosis rate from 16.33 ± 0.32 to 11.57 ± 0.56 (Fig. S5A). Furthermore, as described above, the original expression level of ZHX2 was higher in SMMC7721 cells than in HepG2 cells, and I-125 induced a higher apoptosis rate in SMMC7721 cells, indicating that ZHX2 enhanced the radiosensitivity of HCC cells to I-125. Similar results were obtained in the TUNEL assay (Fig. 6B and Fig. S5B). The CCK-8 assay and EdU staining results (Fig. S6A) showed that, compared to I-125 treatment alone, overexpression of ZHX2 combined with I-125 treatment significantly decreased cell growth in HepG2 cells, whereas the I-125-induced decrease in cell proliferation was attenuated via downregulation of ZHX2 in SMMC7721 cells (Fig. 6C, D). Next, cell migration ability was detected by performing wound healing (Fig. 6E) and transwell assays (Fig. 6F). Although I-125 significantly reduced the wound closure rate, ZHX2 upregulation boosted this effect in HepG2 cells, whereas ZHX2 downregulation attenuated this effect in SMMC7721 cells (Fig. S7A). Consistently, the overexpression or knockdown of ZHX2 enhanced or reversed the I-125 induced anti-migration effect in HCC cells (Fig. S7B). Moreover, the I-125-induced G2/M phase cell cycle arrest rate was enhanced when ZHX2 was overexpressed, increasing from 20.72 ± 0.85 to 28.92 ± 0.66. However, downregulation of ZHX2 reversed the increase in the I-125-induced cell cycle arrest rate, ranging from 21.99 ± 0.52 to 17.29 ± 0.20 (Fig. 6G and Fig. S6B). The role of ZHX2 in the I-125-induced anticancer effects was further verified in vivo. As shown in Fig. 6H, compared to the control group, I-125 significantly inhibited tumor volume, which could be compromised when ZHX2 was downregulated; the change in tumor weight showed a similar result (Fig. 6I). Generally, ZHX2 enhanced I-125-induced anticancer effects on HCC cells.

### GADD34 is a transcriptional target of ZHX2

**Fig. 7:**
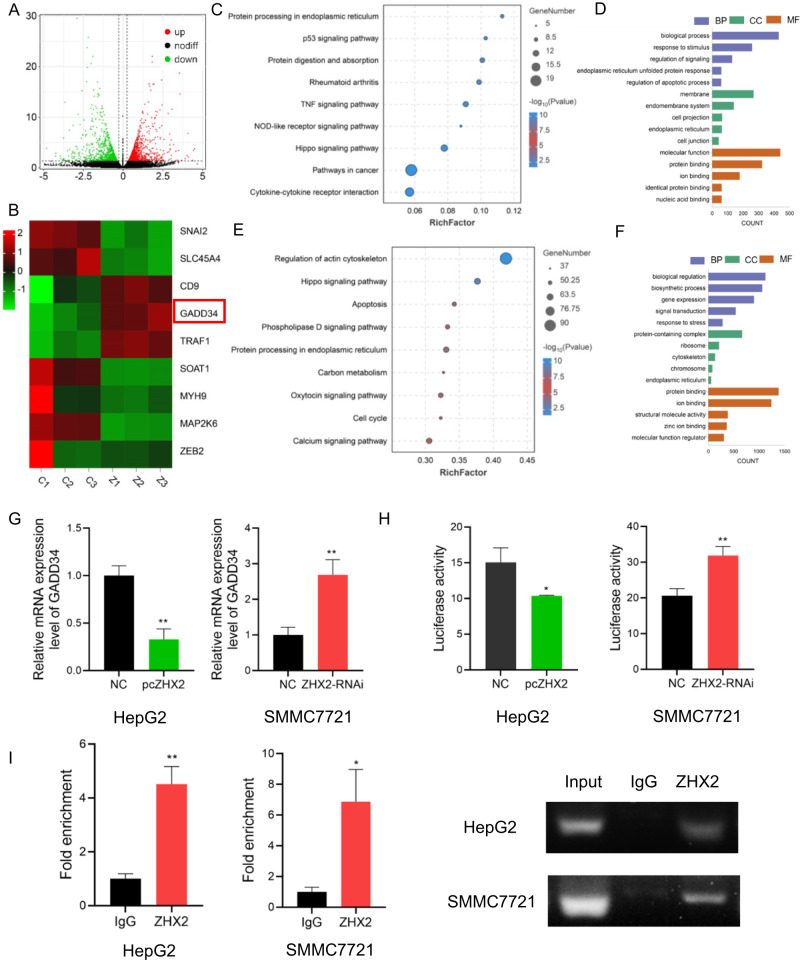
GADD34 is a transcriptional target of ZHX2. **A**, **B** Based on the results of RNA-seq performed with SMMC7721 cells transfected with control-RNAi or ZHX2-RNAi, the volcano map and heatmap show the differentially expressed mRNAs. **C**, **D** The KEGG pathway enrichment and GO annotation of the differentially expressed mRNAs in RNA-seq. **E**, **F** The KEGG pathway enrichment and GO annotation of the differentially expressed mRNAs in ChIP-seq performed with SMMC7721 cells transfected with control-RNAi or ZHX2-RNAi. **G** qRNA for detecting the GADD34 expression level in HepG2 and SMMC7721 cells after transfected with pcZHX2 or ZHX2-RNAi. **H** The dual luciferase reporter assay for detecting GADD34 promoter activity in HepG2 and SMMC7721 cells after transfected with pcZHX2 or ZHX2-RNAi. **I** The ChIP assay to detect ZHX2 occupancy on the promoter of GADD34 in HepG2 and SMMC7721 cells. The quantification of qRT-PCR (left) and semiquantitative images (right) are shown. The data are presented as the mean ± SD. *P < 0.05, **P < 0.01.

To further clarify the molecular mechanism underlying the role of ZHX2 in regulating the I-125-induced anticancer effects, RNA-seq and chromatin immunoprecipitation (ChIP)-seq were performed and potential downstream genes of ZHX2 were identified. RNA-seq was performed on SMMC7721 cells transfected with NT-RNAi or ZHX2-RNAi. The results demonstrated that 553 genes had altered expressions; 284 were upregulated and 269 were downregulated (Fig. 7A). Cluster analysis demonstrated differences in the expression of 10 genes between the control and ZHX2-RNAi groups, including GADD34, a key regulator of ER stress, whose expression level was significantly increased (Fig. 7B). KEGG and Gene Ontology (GO) analyses revealed protein processing in the ER (hsa04141), ER unfolded protein response (GO:0030968), and ER (GO:0005783) (Fig. 7C, D). For ChIP-seq, 3.4 × 10^7^ reads were mapped to the reference genome (Fig. S8A, B). A total of 9325 peaks were detected; over 30% of the peaks were identified in the promoter regions, which correlated with the function of ZHX2 as a transcription factor. Peaks were distributed in the untranslated regions (1.35%), exons (1.78%), introns (36.98%), downstream regions (1.13%), and distal intergenic regions (28.65%). The binding sites for ZHX2 were centered around the gene transcription start sites, especially ≤2 kb (Fig. S8C). Further evaluation of the genes related to the repeat peaks revealed significant enrichment in KEGG pathways, especially including protein processing in ER (hsa04141), the cell cycle (hsa04110), and apoptosis (hsa04215); significantly enriched GO terms included response to stress (GO:0006950), ER (GO:0005783), and protein binding (GO:0005515; Fig. 7E, F). Generally, KEGG and GO analyses revealed enriched protein processing in the ER and ER unfolded protein responses in RNA-seq and ChIP-seq, indicating that ZHX2 was closely related to ER stress. More importantly, RNA-seq identified the ER stress negative regulator, GADD34, as a potential downstream target gene of ZHX2. Taken together, our results led us to the hypothesis that ZHX2 enhances the induced anticancer effects of I-125 on HCC cells through ER stress regulated by GADD34, which has not yet been proven. To confirm this hypothesis, qRT-PCR was performed on HepG2 and SMMC7721 cells, which revealed a negative regulatory relationship between ZHX2 and GADD34 (Fig. 7G). Furthermore, a dual-luciferase reporter assay indicated that ZHX2 binds to the promoter region of GADD34, confirming a direct association (Fig. 7H). Moreover, qRT-PCR showed that the mRNA expression of GADD34 was higher in the ZHX2 group than in the IgG group, confirming the binding effect between the ZHX2 protein and GADD34 DNA (Fig. 7I). Hence, ZHX2 transcriptionally inhibits GADD34 expression.

### ZHX2 promotes I-125-induced anticancer effect in HCC via regulating GADD34

**Fig. 8:**
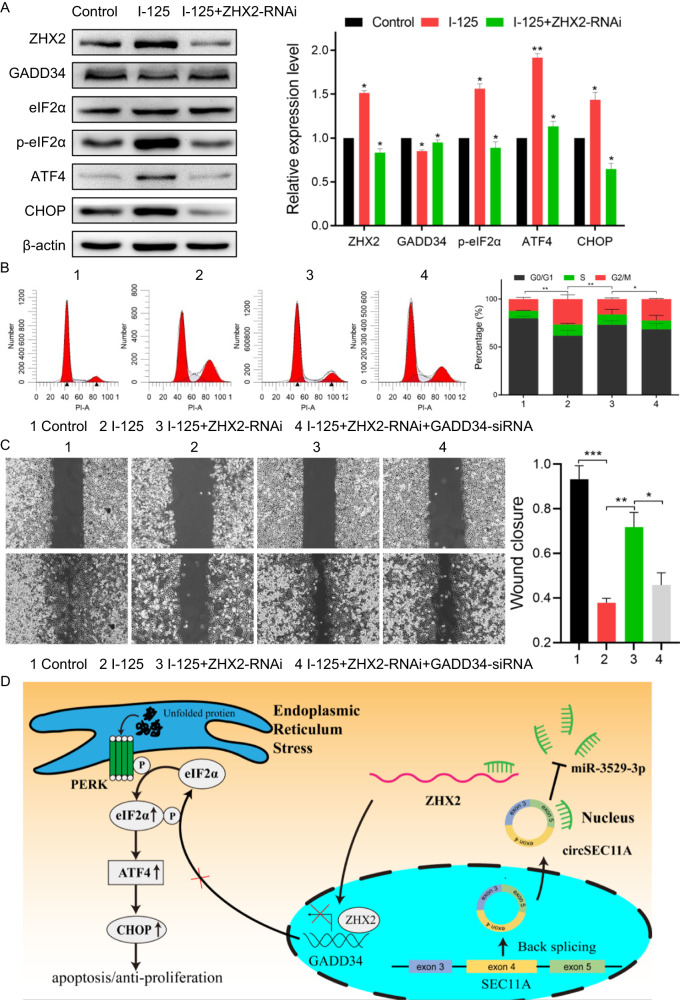
ZHX2 promotes I-125-induced anticancer effect in HCC via regulating GADD34. **A** The western blot was performed to detect the expression level of ZHX2, GADD34, eIF2α, ATF4, and CHOP in SMMC7721 cells transfected with NT-RNAi or ZHX2-RNAi and I-125. **B**, **C** The flow cytometry and wound healing to detect cell cycle and cell migration ability treated with I-125, ZHX2-RNAi, and GADD34-siRNA in SMMC7721 cells. **D** Schematic illustration of circSEC11A/miR-3529-3p/ZHX2/GADD34 axis in regulating I-125-induced anti-cancer effect in HCC. The data are presented as the mean ± SD. *P < 0.05, **P < 0.01, ***P < 0.001.

As mentioned above, a scientific hypothesis was proposed that ZHX2 enhances the I-125-induced anticancer effects on HCC through ER stress regulated by GADD34. Several assays were performed to verify the direct regulatory relationship between ZHX2 and GADD34. Next, the protein and mRNA expression levels of GADD34 and ER stress pathway-related genes, including eIF2α, p-eIF2α, ATF4, and CHOP, were detected (Fig. 8A and Fig. S9A). The results showed that ZHX2 negatively regulated GADD34, while positively regulating the p-eIF2α-ATF4-CHOP ER stress pathway. To better investigate the role of GADD34 in the ZHX2-mediated regulation of I-125-induced anticancer effects, GADD34 was downregulated in SMMC7721 cells via transfection with siRNA. The regulatory effect of siRNA was verified using western blotting, and the inhibitory effect of siRNA-1 was found to be the strongest (Fig. S9B). Furthermore, cell cycle and wound healing assays were performed to investigate if GADD34 could reverse the function of ZHX2 in regulating I-125-induced anticancer effects. As shown in Fig. 8B, compared to the control group, the G2/M phase rate was increased in the I-125 group from 12.36 ± 1.02 to 26.61 ± 2.54, which could be reversed to 16.13 ± 0.71 by downregulating ZHX2. More interestingly, the attenuation effect of ZHX2 could be reversed by GADD34, which increased the G2/M phase rate to 22.5 ± 0.32. Furthermore, I-125 decreased the wound closure rate from 0.932 ± 0.06 to 0.37 ± 0.02. However, this decrease could be attenuated to 0.71 ± 0.06 via downregulating ZHX2, which could also be reversed to 0.45 ± 0.05 when the expression level of GADD34 was inhibited (Fig. 8C). The schematic illustration in Fig. 8D better demonstrates the scientific hypotheses and molecular regulatory relationship in this study. Taken together, these results suggest that ZHX2 promotes the induced anticancer effects of I-125-on HCC cells by regulating GADD34.

## Discussion

I-125 radioactive seed implantation is used for the local treatment of HCC. In clinical practice, it is mostly used in patients with HCC who are unable to undergo surgery, are insensitive to traditional radiotherapy, have poor control after TACE, and are at high risk of ablation in specific parts such as the diaphragm and gallbladder [19, 20]. However, owing to differences in I-125 radiosensitivity among patients, the local tumor control rate in radioinsensitive patients is not ideal. Therefore, elucidating the molecular mechanism of action of I-125 in the treatment of HCC has become a focus of clinical research and is an urgent problem to be solved in clinical practice. Our previous study revealed the role of the eIF2α-induced ER stress pathway in regulating the I-125-induced anticancer effects on HCC [21]. However, the upstream regulatory mechanisms underlying this pathway remain unknown. For HCC, circRNA has been reported to be involved in a variety of biological processes [22]. However, few studies have focused on I-125 radioactive seeds. Moreover, although ZHX2 has been identified as a tumor suppressor gene in HCC and can promote chemotherapy sensitivity in HCC, its role in regulating the l-125-induced anticancer effects remains unclear [23–25]. Based on our previous study and the potential role of circRNAs and ZHX2 in regulating radiosensitivity, RNA-seq was performed to identify differentially expressed circRNAs and mRNAs after treatment with I-125. Furthermore, ChIP-seq was performed to identify the potential targets of ZHX2, which is also related to the ER stress pathway. Finally, we discovered the essential role of the circSEC11A/miR-3529-3p/ZHX2/GADD34 axis in regulating the I-125-induced anticancer effects on HCC.

CircRNAs can modify the phenotype and determine the fate of tumor cells through molecular regulatory mechanisms such as molecular sponges, binding proteins, and self-coding short peptides [26]. More importantly, studies have reported the role of circRNAs in the regulation of radiation-induced anticancer effects [27, 28]. Wang et al. found that low-dose radiation-induced circMETRN plays an oncogenic role in glioblastoma and acts as a sponge for miR-4709-3p to regulate radiosensitivity [29]. In addition, a study based on clinical samples revealed that circ_0071662 was not only downregulated in and functional as a biomarker for the diagnosis of HCC, but also had a close relationship with radioresistance [30]. However, the role of circRNAs in regulating the I-125-induced anticancer effects has not yet been elucidated. In the current study, RNA-seq analysis of I-125-treated HCC cells showed that circSEC11A was significantly upregulated. Cell function experiments showed that knockdown of circSEC11A attenuated the I-125-induced anticancer effects on HCC in vivo and in vitro. Furthermore, due to circSEC11A is located at the cytoplasm, the ceRNA network has been suggested as the main molecular mechanism of circSEC11A. In addition, considering that ZHX2 is significantly upregulated in HCC cells treated with I-125, the circSEC11A/miR-3529-3p/ZHX2 axis was established using several bioinformatics websites to predict the interaction between circRNA, miRNA, and mRNA, which was verified using RIP, RNA pull-down, and dual-luciferase reporter assays. In addition, FISH confirmed the colocalization of circSEC11A and miR-3529-3p. Experiments demonstrated that miR-3529-3p reversed the function of circSEC11A in the I-125-induced anticancer effects.

The ZHX2 transcription factor is a member of the ZHX family and is involved in the transcriptional regulation of key genes closely related to the occurrence and development of various tumors, including HCC, myeloma, and renal cancer [31–33]. Studies have shown that ZHX2 can negatively regulate AFP, GPC3, and H19 [33, 34]. In liver cancer tissues, the expression of ZHX2 is significantly reduced owing to the methylation of the ZHX2 promoter, which is positively correlated with the degree of tumor differentiation [35]. ZHX2 can bind to the *CCNA2* and *CCNE1* regions in the promoters of cyclins A and E to inhibit their transcription [15]. Studies have shown that ZHX2 is negatively correlated with the expression of MDR1 in liver cancer tissue and that ZHX2 can inhibit the expression of *MDR1* at the transcriptional level and promote the sensitivity of liver cancer to chemotherapy drugs [11]. Although studies have confirmed the regulatory role of ZHX2 as a transcriptional factor in chemotherapeutic drug sensitivity, the relationship between ZHX2 and radiotherapy has not been yet clarified, and it is essential to investigate the upstream regulatory mechanism of ZHX2. In this study, we used RNA-seq to identify the differential expression levels of ZHX2 after treatment with I-125 and confirmed the I-125-induced the upregulation of ZHX2 in vivo and in vitro. Furthermore, cell and animal functional experiments showed that ZHX2 enhanced the I-125-induced anticancer effects on HCC.

The ER is an important intracellular organelle; it is the main base for intracellular protein synthesis and is an important site for protein post-translational modification, folding, and transport [36]. Under the conditions of radiation exposure, an increase in the levels of unfolded proteins occurs when the processing capacity of the ER is exceeded, leading to ER stress [37]. The eIF2α-induced pathway is one of three ER stress pathways, which are activated through the phosphorylation of eIF2α at serine 51 [37]. Our previous study indicated that the eIF2α-induced ER stress pathway was significantly upregulated in I-125-treated HCC cells [21]. Knockdown of this pathway reversed the I-125-induced anticancer effects on HCC in vivo and in vitro. Furthermore, Yang et al. found that the eIF2α-induced ER stress pathway could be upregulated by I-125 in a dose-dependent manner and relieved the I-125-induced radiation myelitis in neuron cells, which was consistent with our previous study [38]. In this study, we identified GADD34, which dephosphorylated eIF2α and negatively regulated the eIF2α-induced ER stress pathway, as a potential transcriptional target of ZHX2 through RNA- and ChIP-seq performed with NT-RNAi and ZHX2-RNAi SMMC7721 cells [39]. Furthermore, dual-luciferase reporter and chromatin immunoprecipitation assays were performed to prove that ZHX2 could bind to the promoter region of GADD34. More importantly, the results of western blotting showed that ZHX2 negatively regulated GADD34 and positively regulated the eIF2α-induced ER stress pathway. In addition, cell function experiments suggested that GADD34 could reverse the function of ZHX2 in the I-125-induced anticancer effects on HCC.

In this study, RNA-seq was performed to identify differentially expressed circRNAs, miRNAs, and mRNAs in HCC cells after treatment with I-125. We first revealed the role of circSEC11A and ZHX2 in the I-125-induced anticancer effects on HCC. Furthermore, based on the location of circSEC11A in the cytoplasm, we predicted that miR-3529-3p is a sponge target of circSEC11A, which regulates ZHX2.

In addition, we performed RIP, RNA pull-down, dual-luciferase reporter, and FISH assays to detect anticancer phenotypes, including apoptosis, proliferation, and metastasis, to confirm the interactions between the circSEC11A/miR-3529-3p/ZHX2 axis. To further investigate the molecular regulatory mechanism of ZHX2 based on the enrichment analysis results of RNA- and ChIP-seq, ER stress-related pathways were commonly presented in GO and KEGG analyses. Interestingly, GADD34, a negative regulator of ER stress, has been identified as a target of ZHX2, which forms a bridge between ZHX2 and ER stress. Altogether, our study demonstrates the role of the circSEC11A/miR-3529-3p/ZHX2/GADD34 axis in the I-125-induced anticancer effect on HCC. However, our study has some limitations. First, we only verified the function of circRNAs as miRNA sponges. Other mechanisms of circRNAs, such as translation and direct binding to proteins, have not been investigated. Second, clinical sample research was not conducted in this study. Although samples are difficult to obtain after I-125 treatment because of ethical limitations, samples before treatment should be collected and used to evaluate the diagnosis and prognosis of I-125 treatment. Third, the impact of circSEC11A on the phonotype of ERS was not investigated, which will be demonstrated in our future work.

In summary, we confirmed that circSEC11A and ZHX2 were not only upregulated in I-125-treated HCC cells, but that they also enhanced the I-125-induced anticancer effects on HCC. Mechanistically, circSEC11A competitively binds to miR-3529-3p and reduces the inhibitory effect of miR-3529-3p on ZHX2. In addition, ZHX2 positively regulates the eIF2α-induced ER stress pathway through transcriptionally inhibiting GADD34. In conclusion, our findings elucidate the mechanism of I-125-induced anticancer effects in HCC and lay the foundation for the wider clinical application of I-125 treatment for HCC.

## Materials and methods

### Cell lines, transfection, and I-125 irradiation model

The HepG2, QSG-7701, Huh7, and SMMC7721 cell lines were purchased from the Chinese Academy of Sciences (Shanghai, China) and were identified by STR profiling. The cells were incubated in a humidified 5% CO_2_ incubator at 37 °C and were supplemented with 10% fetal calf serum (Gibco, Waltham, MA USA). The HepG2 and QSG-7701 cells were cultured in Dulbecco’s modified Eagle’s medium (Corning, Inc., Corning, NY, USA); Huh7 and SMMC7721 cells were cultured in RPMI 1640 (Corning, Inc., Corning, NY, USA). A short interfering RNA (siRNA) for circSEC11A, miR-3529-3p mimics, miR-3529-3p inhibitor, ZHX2, and GADD34 were designed by RiboBio (Guangzhou, Guangdong, China). The overexpression plasmid for ZHX2 and lentivirus for circSEC11A were purchased from Genechem (Shanghai, China). Cell transfections were performed using lipofectamine 3000 (Invitrogen, CA, USA) according to the manufacturer’s instructions. For the I-125 treatment, the radioactive seeds were purchased from Ningbo Junan Pharmaceutical Technology Company (Ningbo, Zhejiang, China) and were seeded into an in vitro irradiation model. The initial activity and dose rate were 3.0 mCi and 3.412 cGy/h, respectively. The cells with 2 × 10^5^ cells/ml were seeded in a 35 mm-dish and placed into the model.

### Total RNA and genomic DNA extraction

Total RNA was extracted from cell or tissue samples using an RNA-quick Purification Kit (Esunbio, Shanghai, China) as per the manufacturer’s instructions. Genomic DNA (gDNA) was extracted from cell samples using a Genomic DNA Extraction Kit (TIANGEN, Beijing, China). The density and quality of total RNA and gDNA were detected by NanoDrop One (Themo Fisher Scientific, Waltham, USA).

### RNA sequencing and data analysis

RNA-seq was performed by Novogene Corporation. HepG2 cells were treated with or without I-125 to identify circRNAs and miRNAs differentially expressed in response to I-125. To find ZHX2 downstream targets, SMMC7721 cells were transfected with NC-RNAi or ZHX2-RNAi.

HTseq v0.9.1 was used to count the reads mapped to each gene, after which the Fragments Per Kilobase of transcript per Million mapped reads of each gene was calculated based on the length of the gene and read counts mapped to the gene. Genes with an adjusted *P*-value less than 0.05 found by DESeq were considered differentially expressed. GO enrichment analysis of the differentially expressed genes was conducted using the R Goseq package, and *P*-values less than 0.05 were considered significantly enriched. KOBAS software was used to test the statistical enrichment of differentially expressed genes in KEGG pathways.

### Quantitative real-time PCR

Total RNA was reversed transcribed to cDNA using a PrimeScript RT Reagent Kit (TIANGEN, Beijing, China). The relative expression levels of circRNA/mRNA and miRNA were normalized to GAPDH and U6 using the 2^−ΔΔCT^ method. The sequences of related primers are listed in Table S1.

### RNase R and Act-D treatment

For RNase R treatment, total RNA extracted from HepG2 and Huh7 cells was treated with 3 U/μg RNase R (Epicenter, USA) for 30 min at 37 °C. For Act-D treatment, HepG2 and Huh7 cells were treated with 2 μg/ml Act-D for 0, 4, 8, 12, and 24 h. The total RNA was extracted according to the treatment time. The expression level of *SEC11A*, *circSEC11A*, and *GAPDH* were detected by qRT-PCR.

### Fluorescence in situ hybridization

Specific fluorescently labeled circSEC11A and miR-3529-3p FISH probes were designed and produced by GenePharma (Shanghai, China). The procedure was performed according to the manufacturer’s instructions. The images were acquired using confocal laser scanning microscopy LSM800 (Zeiss, Jena, Germany). The sequences of probes are listed in Table S1.

### CCK-8 assay

Cells were seeded in 96-well plates for 0, 24, 48, and 72 h, and CCK-8 reagent (10 μL) was added into each well and incubated for 2 h. The absorbance at 450 nm was detected using a microplate reader (Themo Fisher Scientific, Waltham, USA). Three independent experiments were performed.

### Flow cytometry

Flow cytometry was performed to detect cell cycle and cell apoptosis. For cell cycle, cells were harvest and stained with propidium iodide (PI) and RNase A (Elabscience, Wuhan, China) for 1 h before being fixed with 70% cold ethanol for 2 h. For cell apoptosis, cells were stained with PI and Annexin V-FITC/APC (Elabscience, Wuhan, China). The stained cells were detected using a flow cytometer (Beckman, CA, USA).

### Wound healing and transwell assay

For wound healing assay, 2 × 10^4^ cells were seeded into 12-well plates and the gap was produced using a wound healing model (ibidi, Germany). For the transwell assay, cells were cultured with medium containing 30% fetal calf serum for 48 h. All images were acquired using a microscope (Olympus, Tokyo, Japan). The wound area and cell number were counted using Image J software.

### Animal experiments

A total of 1 × 10^7^ cells/mL were diluted in phosphate-buffered saline and subcutaneously injected in the hind leg of male Balb/c nude mice aged 3–5 weeks. Randomly and blindly divided the mice into each group, with at least 4 mice in each group. The nude mice were purchased from the Animal Research Center of Shandong University. For the I-125 implantation procedure, the tumor was punctured using an 18 G needle (Kakko, Japan) after local anesthesia with lidocaine; the seed (Ningbo Junan Pharmaceutical Technology Company, Nanjing, China) was advanced into the tumor through the needle. Tumor volumes were measured every three days using digital caliper. Twenty-four days later, the mice were sacrificed, and tumors were harvested, measured, lysed for RNA isolation, or fixed in formaldehyde for immunohistochemical (IHC) staining. All mice were housed under specific pathogen-free conditions and euthanized according to regulations formulated by the Shandong University Animal Care Committee.

### Protein extraction and western blot analysis

The protein was extracted from cell samples using RIPA lysis buffer (Beyotime, Beijing, China) and denatured at 95 °C for 5 min. The primary antibodies, including ZHX2 (ab205532), GADD34 (ab9869), eIF2α (ab169528), p-eIF2α (32157), ATF4 (ab184909), and CHOP (ab11419), were purchased form Abcam (Cambridge, UK). The secondary antibodies were purchased from ABclonal (Wuhan, China). The original data of western blot was shown in Supplementary Material.

### IHC analysis

For IHC staining, slides were incubated with antibody against ZHX2 (1:100, A18235, ABclonal) overnight at 4 °C and then incubated with secondary antibody for 30 min. The images were acquired by a Mantra Multispectral imaging system (PerkinElmer, MA, USA) and Nanozoomer Digital Pathology Scanner (Tokyo, Japan).

### TUNEL and EdU staining

After treatment as indicated, the cells were stained with TUNEL reagent (Beyotime Biotechnology, China) for 1 h at 37 °C, and incubated with 4′,6-diamidino-2-phenylindole (DAPI) for 15 min. For EdU staining, a Cell-Light EdU Apollo567 In Vitro Kit (RiboBio, Guangdong, China) was used with 50 μM for 2 h. The images were observed using a fluorescence microscope (Olympus, Tokyo, Japan), and cells were counted using Image J software.

### Dual-luciferase reporter assay

The wild-type and mutant sequence of circSEC11 A, the 3’UTR containing the predicted binding sites with miR-3529-3p and mutant sequence of ZHX2, and the promoter region of GADD34 were cloned into the pGL3-basic vector, separately (Genechem, Shanghai, China). The luciferase activities were detected by a Dual-Luciferase Reporter Gene Assay Kit (Beyotime Biotechnology, Beijing, China).

### Chromatin immunoprecipitation sequencing

ChIP-seq was performed by Novogene Corporation. Library quality was assessed on the Aglient Bioanalyzer 2100 system. Raw data in fastq format were first processed using fastp (version 0.19.11) software. After mapping reads to the reference genome, we used the MACS2 (version 2.1.0) peak calling software to identify regions of IP enrichment over background. A *q*-value threshold of 0.05 was used for all data sets. ChIPseeker was used to retrieve the nearest genes around the peak and annotate the genomic region of the peak. GO enrichment analysis was conducted using the R GOseq package. For KEGG pathway analysis, KOBAS software was used to find the enrichment of peak related genes in KEGG pathways. For ChIP-PCR, the IgG and ZHX2 groups were added with their ChIP grade antibodies (GTX112232, GeneTex, USA) according to the suggested concentration. The immunoprecipitated DNA was detected by qRT-PCR and agarose gel electrophoresis.

### Bioinformatics analysis

The downstream miRNAs of circSEC11A were predicted via circBANK (https://www.circbank.cn) and CSCD (https://gb.whu.edu.cn/CSCD). The potential miRNAs binding to the 3’UTR of ZHX2 were predicted via Targetscan (https://www.targetscan.org) and miRDB (https://mirdb.org).

### RNA immunoprecipitation

RNA immunoprecipitation (RIP) assay was performed using an RNA Immunoprecipitation Kit (Geneseed, Guangzhou, China) according to the manufacturer’s instructions. The cells were incubated with magnetic beads conjugated with anti-AGO2 or anti-IgG antibody (Abcam, Cambridge, UK). After beads were washed and incubated with Proteinase K, RNA was extracted using an RNA-quick Purification Kit (Esunbio, Shanghai, China), and the expression levels of circSEC11A and miR-3529-3p were detected by qRT-PCR.

### RNA pull-down

The specific circSEC11A probe was constructed by GenePharma (Shanghai, China) and the RNA pull-down assay was carried out using a PureBinding^®^ RNA-Protein pull-down Kit (Geneseed, Guangzhou, China) according to the manufacturer’s instructions. Beads with the circSEC11A/miR-3529-3p complex were washed, and the enrichment of circSEC11A and miR-3529-3p was detected by qRT-PCR. The sequence of the circSEC11A probe is shown in Table S1.

### Statistical analysis

Statistical analysis was performed using GraphPad Prism 9 software. Data are reported as the mean ± standard deviation of at least three independent experiments. A Student’s *t* test was utilized to analyze the statistical significance between two groups, and one-way one-way analysis of variance was used for multi-group comparisons. A *P*-value less than 0.05 was considered statistically significant for all experiments; *P < 0.05, **P < 0.01, ***P < 0.001.

### Supplementary information

Supplementary Material

Supplementary Figures

Figure legends for Supplementary figures

Supplementary Table

## Data Availability

Data generated in this study is available from the corresponding author upon reasonable request.

## References

[1] Siegel RL, Miller KD, Fuchs HE, Jemal A (2022). Cancer statistics, 2022. CA Cancer J Clin.

[2] Sung H, Ferlay J, Siegel RL, Laversanne M, Soerjomataram I, Jemal A (2021). Global Cancer Statistics 2020: GLOBOCAN estimates of incidence and mortality worldwide for 36 cancers in 185 Countries. CA Cancer J Clin.

[3] Gourd E (2019). Neoadjuvant radiotherapy improves hepatectomy survival. Lancet Oncol.

[4] Gordan JD, Kennedy EB, Abou-Alfa GK, Beg MS, Brower ST, Gade TP (2020). Systemic therapy for advanced hepatocellular carcinoma: ASCO guideline. J Clin Oncol.

[5] Liu Q, Dai X, Zhou X, Ye F, Zhou Y (2019). Comparison of TACE combined with and without iodine-125 seeds implantation therapy for advanced stage hepatocellular carcinoma: a systematic review and meta-analysis. J BUON.

[6] Zhu ZX, Wang XX, Yuan KF, Huang JW, Zeng Y (2018). Transarterial chemoembolization plus iodine-125 implantation for hepatocellular carcinoma: a systemat ic review and meta-analysis. HPB.

[7] Zhang Y, Zhang X, Xu Y, Fang S, Ji Y, Lu L (2022). Circular RNA and its roles in the occurrence, development, diagnosis of cancer. Front Oncol.

[8] Jagtap U, Anderson ES, Slack FJ (2023). The emerging value of circular noncoding RNA research in cancer diagnosis and treatment. Cancer Res.

[9] Li W, Liu JQ, Chen M, Xu J, Zhu D (2022). Circular RNA in cancer development and immune regulation. J Cell Mol Med.

[10] Shen H, Luan F, Liu H, Gao L, Liang X, Zhang L (2008). ZHX2 is a repressor of alpha-fetoprotein expression in human hepatoma cell lines. J Cell Mol Med.

[11] Ma H, Yue X, Gao L, Liang X, Yan W, Zhang Z (2015). ZHX2 enhances the cytotoxicity of chemotherapeutic drugs in liver tumor cells by repressing MDR1 via interfering with NF-YA. Oncotarget..

[12] Li N, Wu Z, Ma C (2022). ZHX2 in health and disease. Front Oncol.

[13] Fang W, Liao C, Shi R, Simon JM, Ptacek TS, Zurlo G (2021). ZHX2 promotes HIF1alpha oncogenic signaling in triple-negative breast cancer. Elife..

[14] Xie H, Zhou J, Liu X, Xu Y, Hepperla AJ, Simon JM (2022). USP13 promotes deubiquitination of ZHX2 and tumorigenesis in kidney cancer. Proc Natl Acad Sci USA.

[15] Yue X, Zhang Z, Liang X, Gao L, Zhang X, Zhao D (2012). Zinc fingers and homeoboxes 2 inhibits hepatocellular carcinoma cell proliferation and represses expr ession of Cyclins A and E. Gastroenterology..

[16] Yu S, Ruan X, Liu X, Zhang F, Wang D, Liu Y (2021). HNRNPD interacts with ZHX2 regulating the vasculogenic mimicry formation of glioma cells via linc0070 7/miR-651-3p/SP2 axis. Cell Death Dis.

[17] Tan S, Guo X, Li M, Wang T, Wang Z, Li C (2021). Transcription factor Zhx2 restricts NK cell maturation and suppresses their antitumor immunity. J Exp Med.

[18] Tian X, Wang Y, Li S, Yue W, Tian H (2020). ZHX2 inhibits proliferation and promotes apoptosis of human lung cancer cells through targeting p38MA PK pathway. Cancer Biomark.

[19] Chen L, Sun T, Kan X, Chen S, Ren Y, Cao Y (2020). Transarterial chemoembolization combined with iodine-125 seed implantation for patients with hepatoce llular carcinoma: a retrospective controlled study. J Int Med Res.

[20] Li D, Wang WJ, Wang YZ, Wang YB, Li YL (2019). Lobaplatin promotes (125)I-induced apoptosis and inhibition of proliferation in hepatocellular carcinoma by upregulating PERK-eIF2alpha-ATF4-CHOP pathway. Cell Death Dis.

[21] Chen L, Kan X, Sun T, Ren Y, Cao Y, Yan L (2020). Transarterial chemoembolization combined with iodine 125 seeds versus transarterial chemoembolization combined with radiofrequency ablation in the treatment of early- and intermediate-stage hepatocellu lar carcinoma. BMC Gastroenterol.

[22] Wang P, Zhang Y, Deng L, Qu Z, Guo P, Liu L (2022). The function and regulation network mechanism of circRNA in liver diseases. Cancer cell Int.

[23] Yu X, Lin Q, Wu Z, Zhang Y, Wang T, Zhao S (2020). ZHX2 inhibits SREBP1c-mediated de novo lipogenesis in hepatocellular carcinoma via miR-24-3p. J Pathol.

[24] Lin Q, Wu Z, Yue X, Yu X, Wang Z, Song X (2020). ZHX2 restricts hepatocellular carcinoma by suppressing stem cell-like traits through KDM2A-mediated H 3K36 demethylation. EBioMedicine..

[25] Song X, Tan S, Wu Z, Xu L, Wang Z, Xu Y (2018). HBV suppresses ZHX2 expression to promote proliferation of HCC through miR-155 activation. Int J Cancer.

[26] Wang C, Zhou M, Zhu P, Ju C, Sheng J, Du D (2022). IGF2BP2-induced circRUNX1 facilitates the growth and metastasis of esophageal squamous cell carcinoma through miR-449b-5p/FOXP3 axis. J Exp Clin Cancer Res.

[27] Zhang J, Yu Y, Yin X, Feng L, Li Z, Liu X (2022). A Circ-0007022/miR-338-3p/Neuropilin-1 axis reduces the radiosensitivity of esophageal squamous cell carcinoma by activating epithelial-to-mesenchymal transition and PI3K/AKT Pathway. Front Genet.

[28] Zhu S, Chen Y, Ye H, Wang B, Lan X, Wang H (2022). Circ-LARP1B knockdown restrains the tumorigenicity and enhances radiosensitivity by regulating miR-578/IGF1R axis in hepatocellular carcinoma. Ann Hepatol.

[29] Wang X, Cao Q, Shi Y, Wu X, Mi Y, Liu K (2021). Identification of low-dose radiation-induced exosomal circ-METRN and miR-4709-3p/GRB14/PDGFRalpha pathway as a key regulatory mechanism in Glioblastoma progression and radioresistance: functional validation and clinical theranostic significance. Int J Biol Sci.

[30] Wang X, Zhang J, Luo F, Shen Y. Application of CircRNA Circ_0071662 in the diagnosis and prognosis of hepatocellular carcinoma and its response to radiotherapy. Dig dis. (Basel, Switzerland). 2023;3:431–38. 10.1159/000527696 36279855

[31] Maciel NIG, Filiú-Braga LDC, Neves FAR, Rego EM, Lucena-Araujo AR, Saldanha-Araujo F (2021). Low expression of ZHX1 and ZHX2 impacts on the prognosis of chronic lymphocytic leukemia. Biomark Res.

[32] Zhang J, Wu T, Simon J, Takada M, Saito R, Fan C (2018). VHL substrate transcription factor ZHX2 as an oncogenic driver in clear cell renal cell carcinoma. Science..

[33] Luan F, Liu P, Ma H, Yue X, Liu J, Gao L (2014). Reduced nucleic ZHX2 involves in oncogenic activation of glypican 3 in human hepatocellular carcinoma. Int J Biochem Cell Biol.

[34] Lv Z, Du Y, Wen J (2013). The methylation of ZHX2 gene promoter enhances AFP gene expression in hepatocellular carcinoma. Xi Bao Yu Fen Zi Mian Yi Xue Za Zhi.

[35] Lv Z, Zhang M, Bi J, Xu F, Hu S, Wen J (2006). Promoter hypermethylation of a novel gene, ZHX2, in hepatocellular carcinoma. Am J Clin Pathol.

[36] Chen X, Cubillos-Ruiz JR (2021). Endoplasmic reticulum stress signals in the tumour and its microenvironment. Nat Rev Cancer.

[37] King AP, Wilson JJ (2020). Endoplasmic reticulum stress: an arising target for metal-based anticancer agents. Chem Soc Rev.

[38] Yang Z, Xu Y, Xu L, Maccauro G, Rossi B, Chen Y (2013). Regulation of autophagy via PERK-eIF2alpha effectively relieve the radiation myelitis induced by iodine-125. PLoS One.

[39] Lebeaupin C, Vallee D, Hazari Y, Hetz C, Chevet E, Bailly-Maitre B (2018). Endoplasmic reticulum stress signalling and the pathogenesis of non-alcoholic fatty liver disease. J Hepatol.

