# Supplementary material for: Characterization of circSEC11A as a novel regulator of Iodine-125 radioactive seed-induced anticancer effects in hepatocellular carcinoma via targeting ZHX2/GADD34 axis
Source: Cell Death Discov. 2023 Aug 10;9:294. doi: 10.1038/s41420-023-01593-w (PMC10415397; doi:10.1038/s41420-023-01593-w)
Supplement: Supplementary file 1 — Supplementary Material [file 41420_2023_1593_MOESM1_ESM.pdf]

Figure 4D

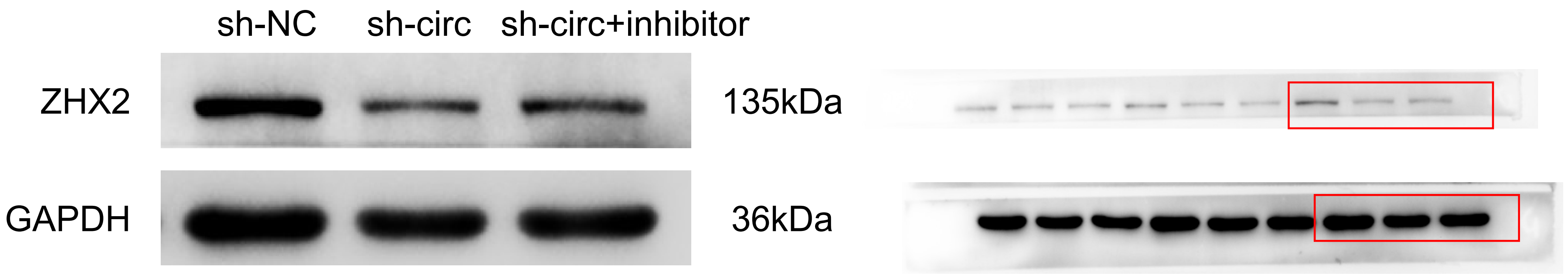

Figure 5D

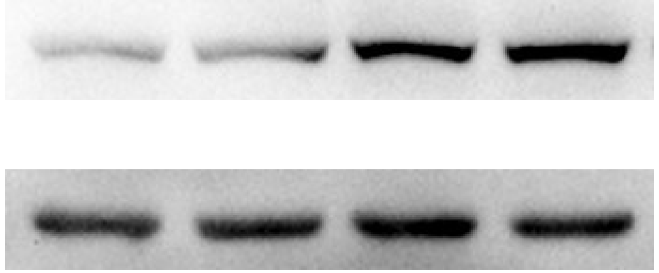

ZHX2

β-actin

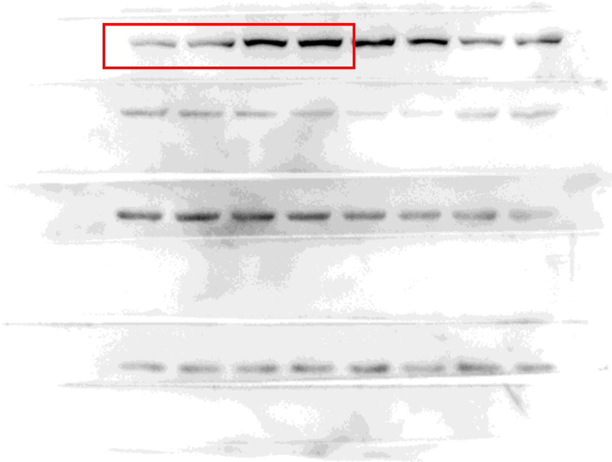

ZHX2

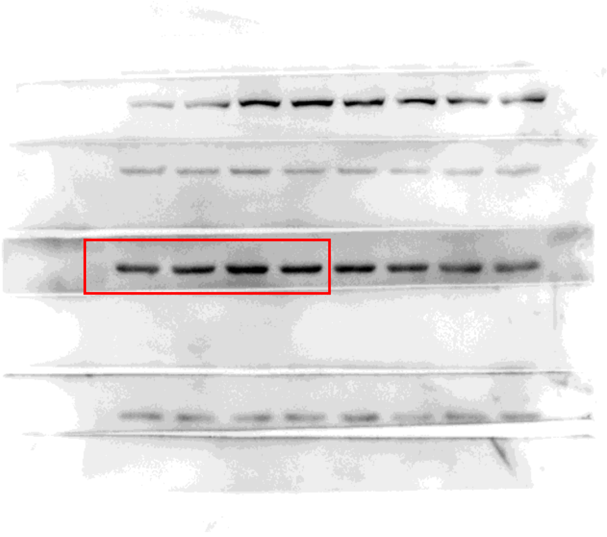

β-actin

Figure 5F

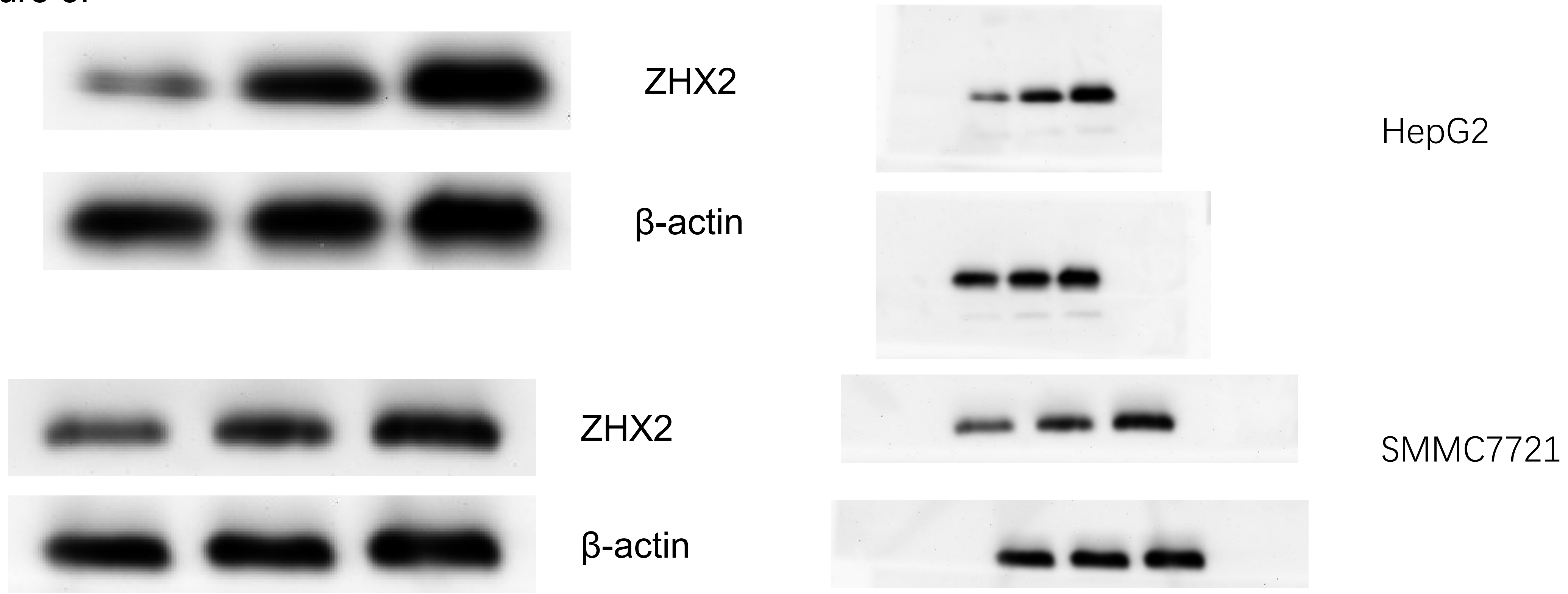

Figure 5H

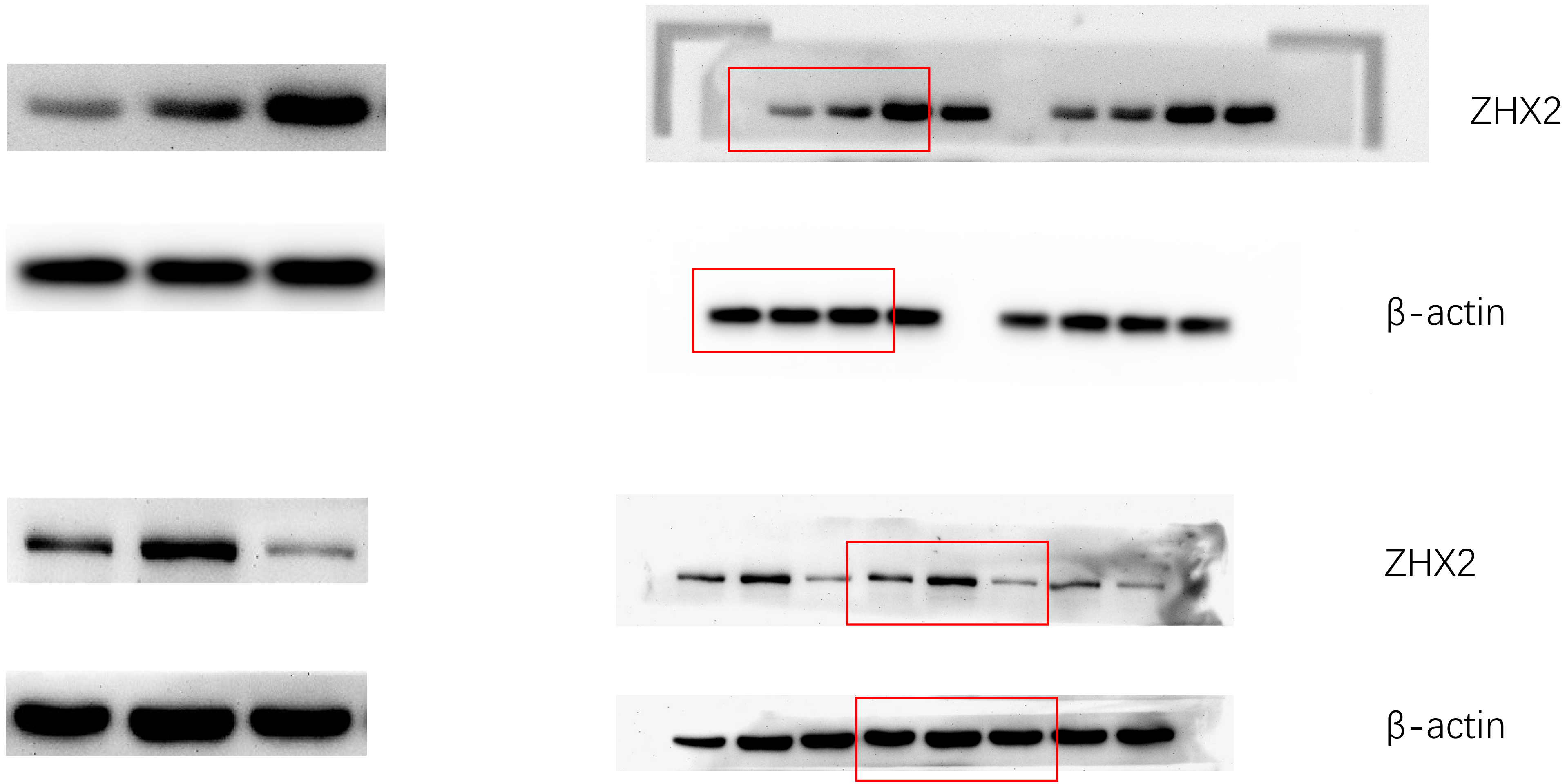

Figure 8A

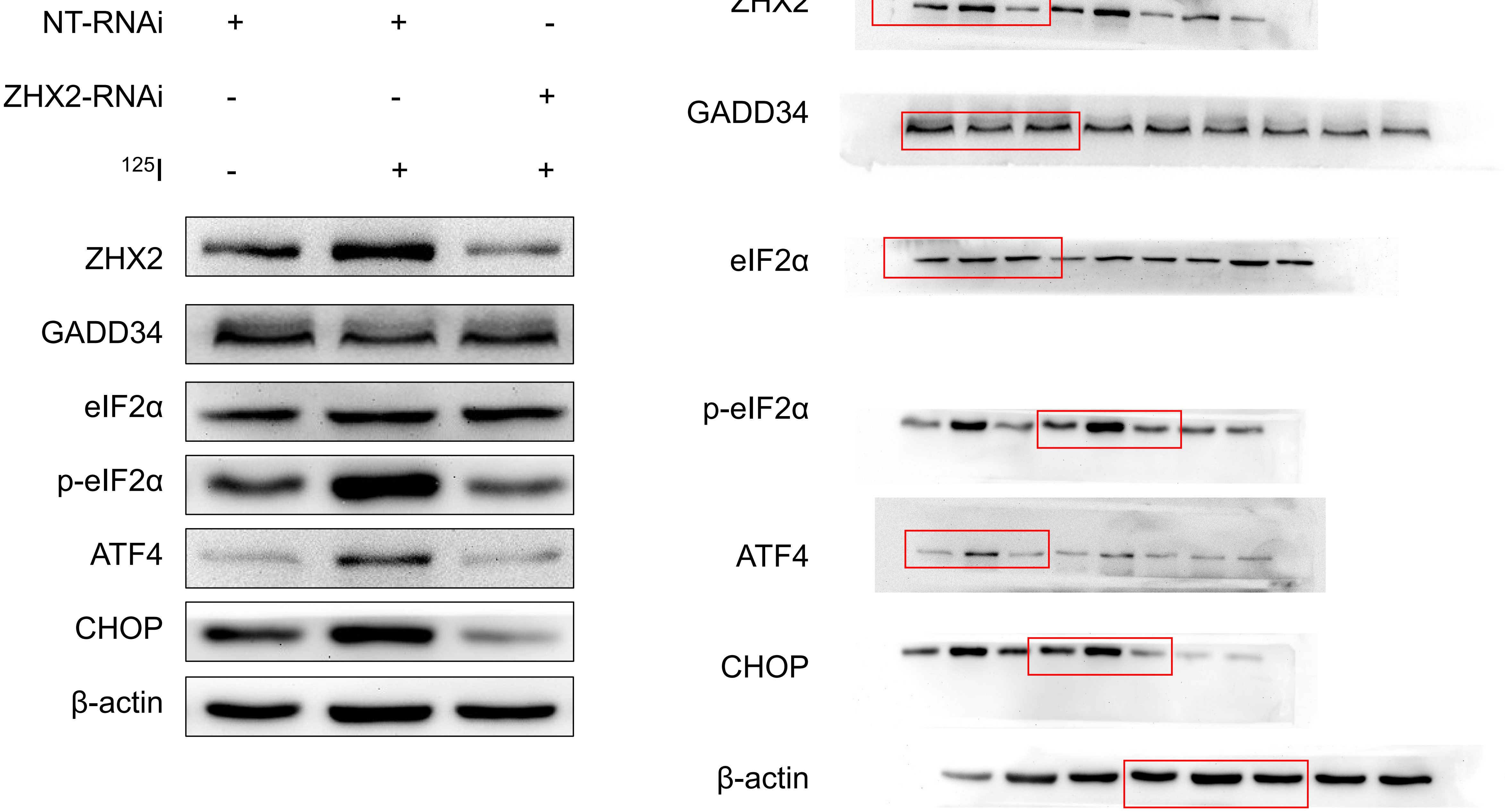
NT-RNAi + + -
ZHX2-RNAi - - +
125I - + +
ZHX2
GADD34
eIF2α
p-eIF2α
ATF4
CHOP
β-actin
ZHX2
GADD34
eIF2α
p-eIF2α
ATF4
CHOP
β-actin
