# Supplementary material for: Characterization of circSEC11A as a novel regulator of Iodine-125 radioactive seed-induced anticancer effects in hepatocellular carcinoma via targeting ZHX2/GADD34 axis
Source: Cell Death Discov. 2023 Aug 10;9:294. doi: 10.1038/s41420-023-01593-w (PMC10415397; doi:10.1038/s41420-023-01593-w)
Supplement: Supplementary file 2 — Supplementary Figures [file 41420_2023_1593_MOESM2_ESM.pdf]

Figure S1

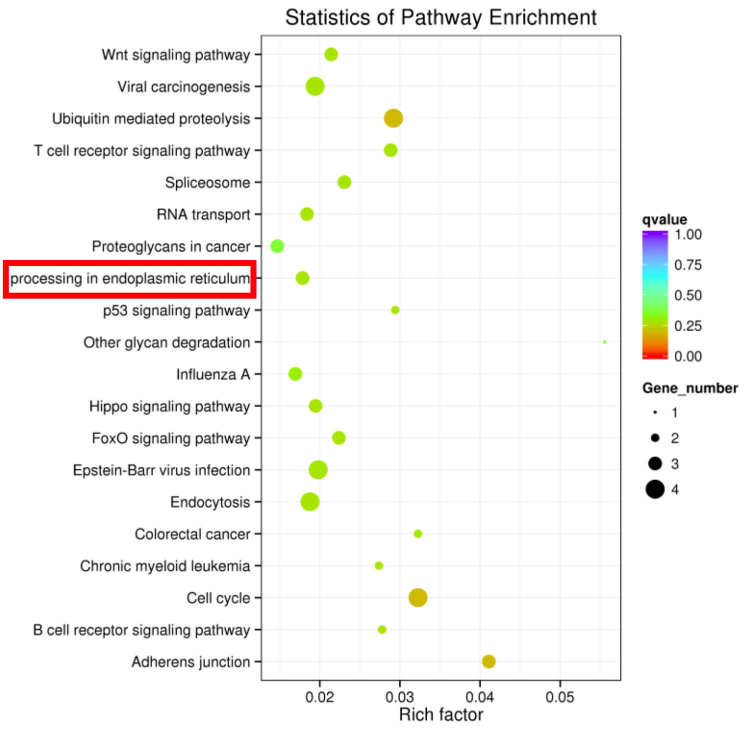

Statistics of Pathway Enrichment
Wnt signaling pathway
Viral carcinogenesis
Ubiquitin mediated proteolysis
T cell receptor signaling pathway
Spliceosome
RNA transport
Proteoglycans in cancer
processing in endoplasmic reticulum
p53 signaling pathway
Other glycan degradation
Influenza A
Hippo signaling pathway
FoxO signaling pathway
Epstein-Barr virus infection
Endocytosis
Colorectal cancer
Chronic myeloid leukemia
Cell cycle
B cell receptor signaling pathway
Adherens junction
0.02
0.03
0.04
0.05
Rich factor
qvalue
1.00
0.75
0.50
0.25
0.00
Gene_number
1
2
3
4

Figure S2

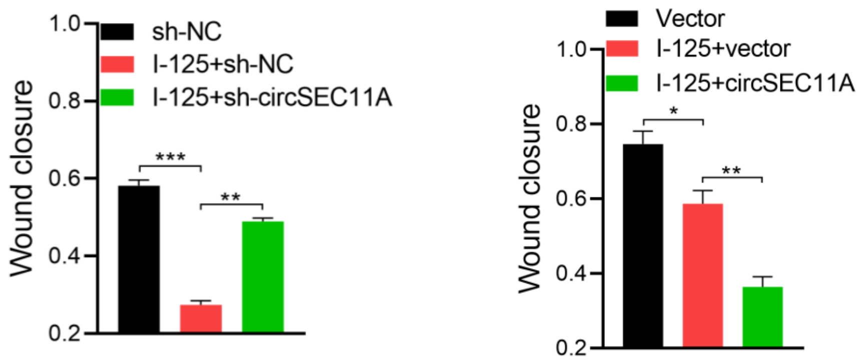
1.0
0.8
0.6
0.4
0.2
Wound closure
sh-NC
I-125+sh-NC
I-125+sh-circSEC11A
***
**
Vector
I-125+vector
I-125+circSEC11A
1.0
0.8
0.6
0.4
0.2
Wound closure
*
**

Figure S3

A

circSEC11A WT 5' AUACGAGUGGGAGAAAUUGUUGUU 3'

miR-3529-3p 3' ACCUUCUGAUCACUAAAACAACAA 5'

circSEC11A Mut 5' AUACGAGUGGGAGAAAGGUGGUGG 3'

B

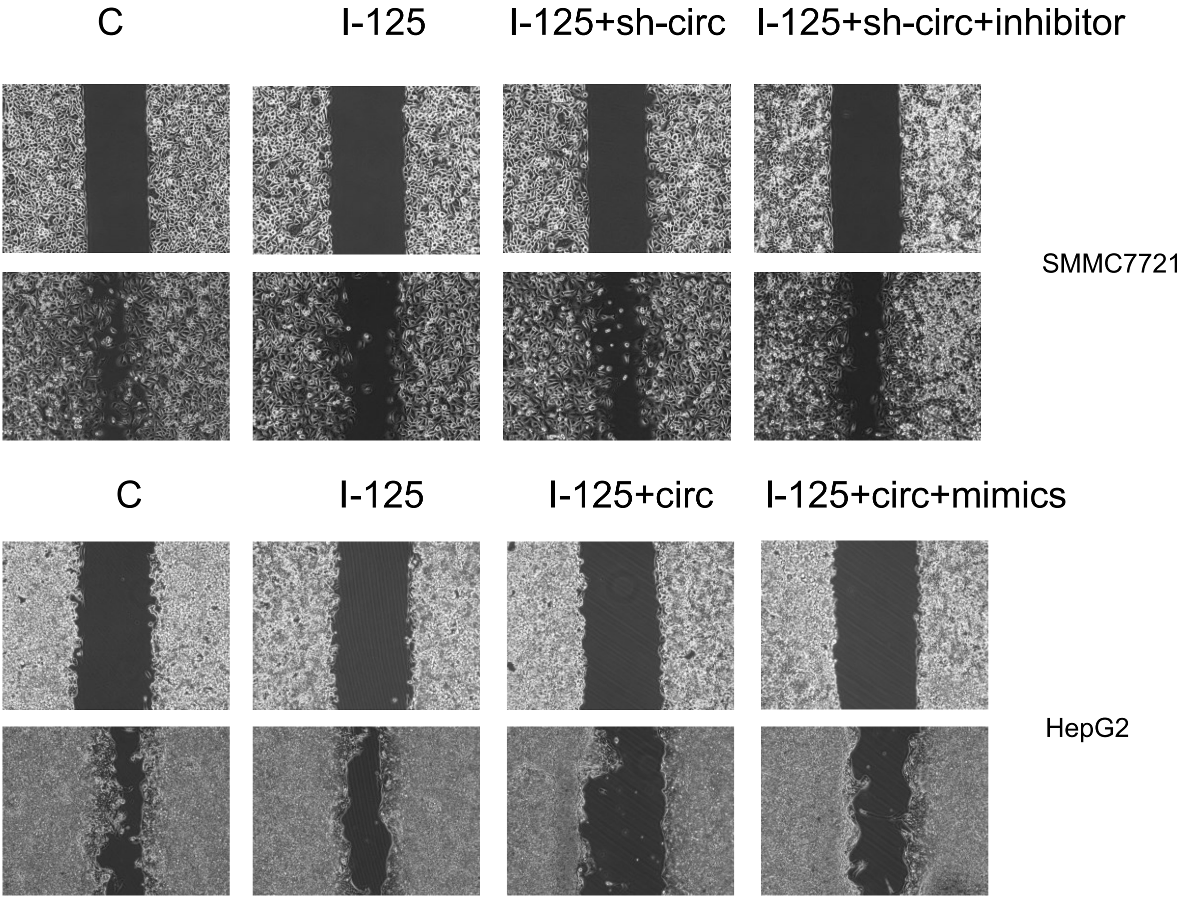

Figure S4

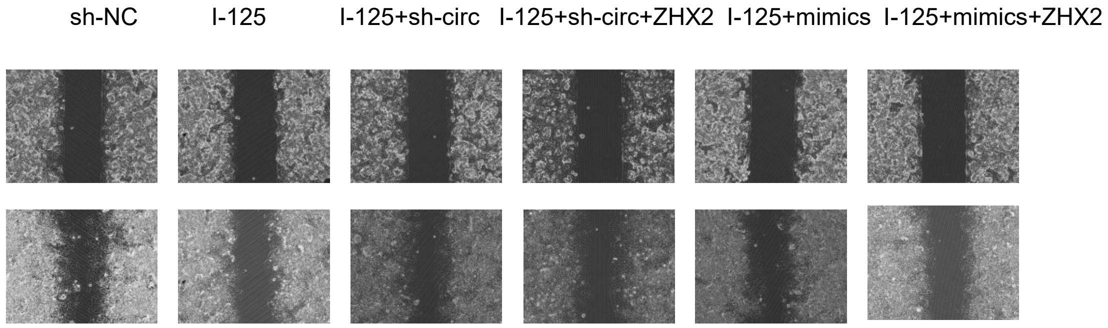

sh-NC
I-125
I-125+sh-circ
I-125+sh-circ+ZHX2
I-125+mimics
I-125+mimics+ZHX2

Figure S5

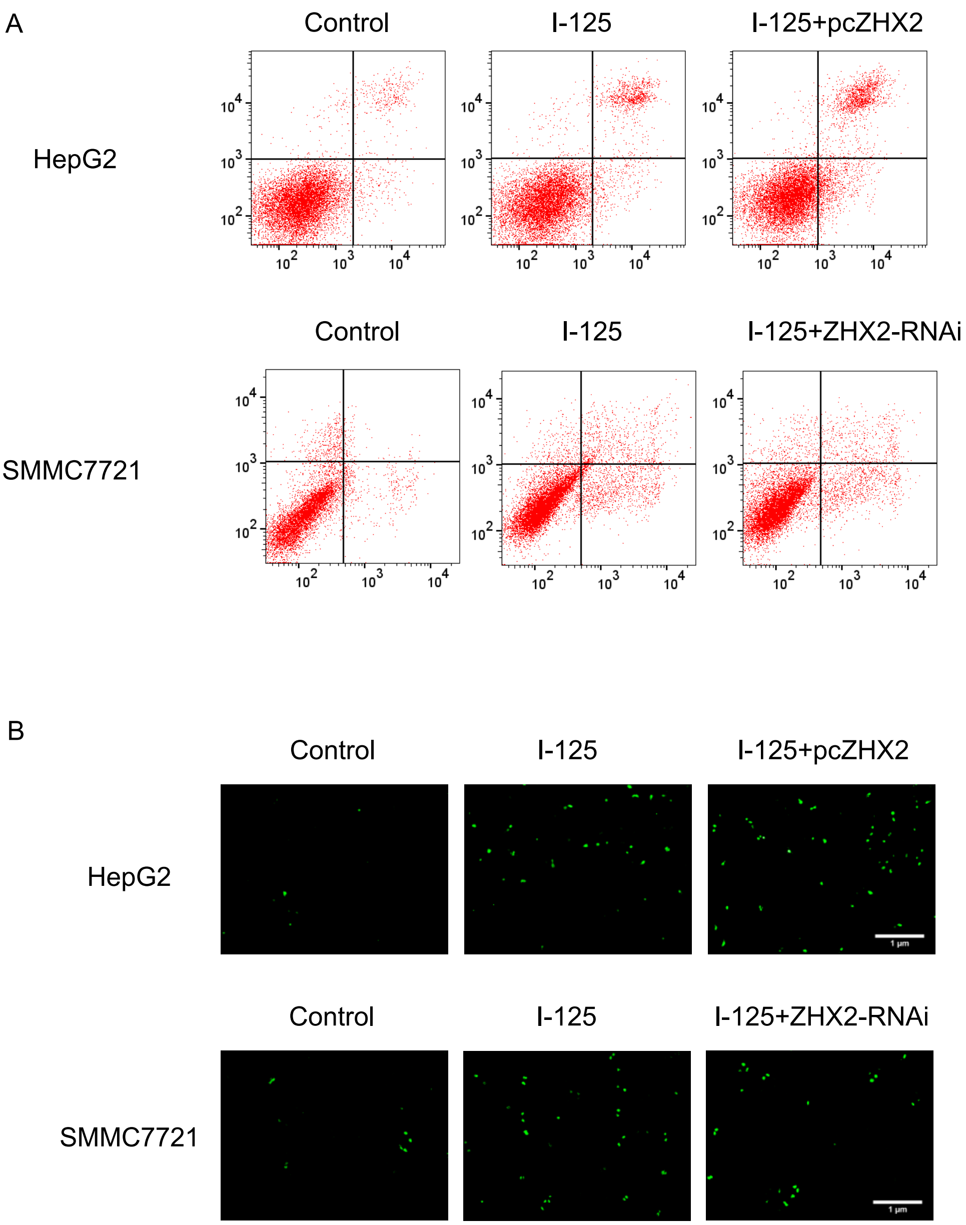
A
Control
I-125
I-125+pcZHX2
HepG2
Control
I-125
I-125+ZHX2-RNAi
SMMC7721
B
Control
I-125
I-125+pcZHX2
HepG2
Control
I-125
I-125+ZHX2-RNAi
SMMC7721

Figure S6

A

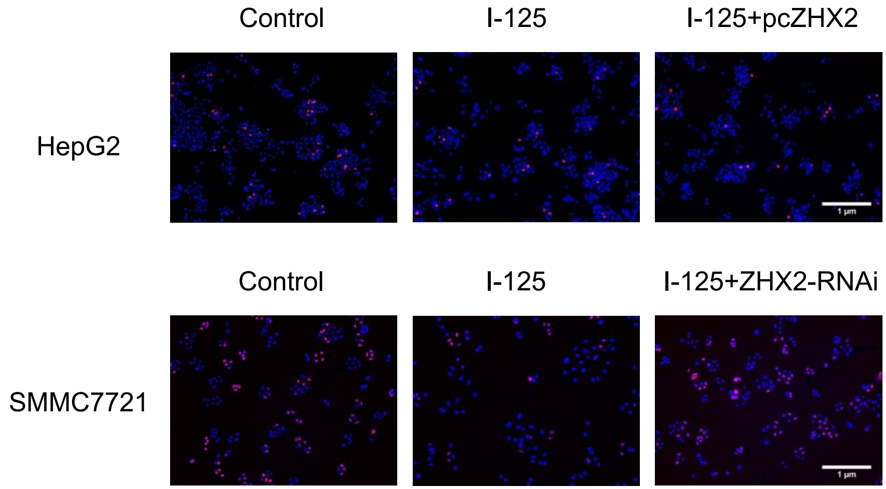
Control
I-125
I-125+pcZHX2
HepG2
1 μm
Control
I-125
I-125+ZHX2-RNAi
SMMC7721
1 μm

B

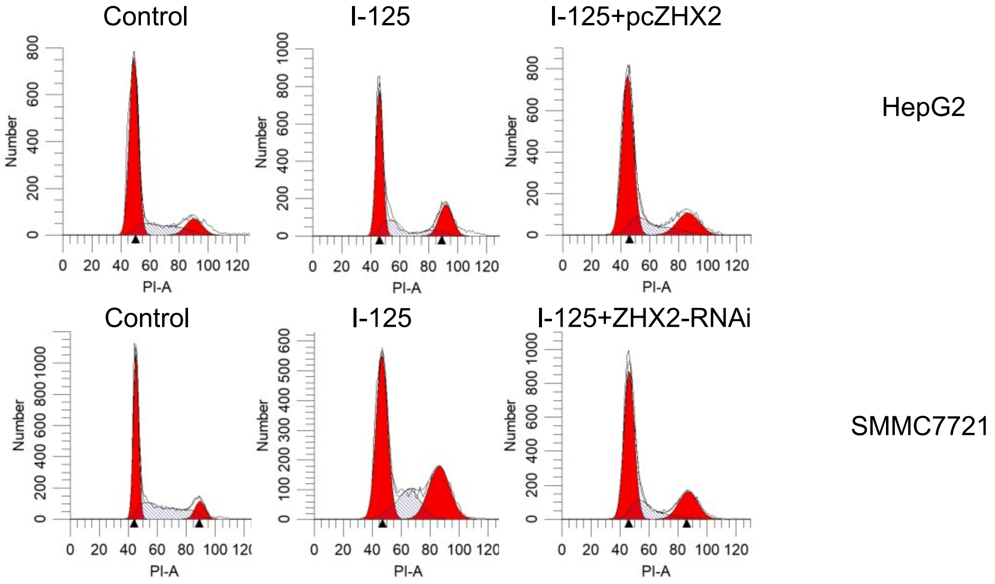
Control
I-125
I-125+pcZHX2
HepG2
Control
I-125
I-125+ZHX2-RNAi
SMMC7721
Number
PI-A
0 20 40 60 80 100 120

Figure S7

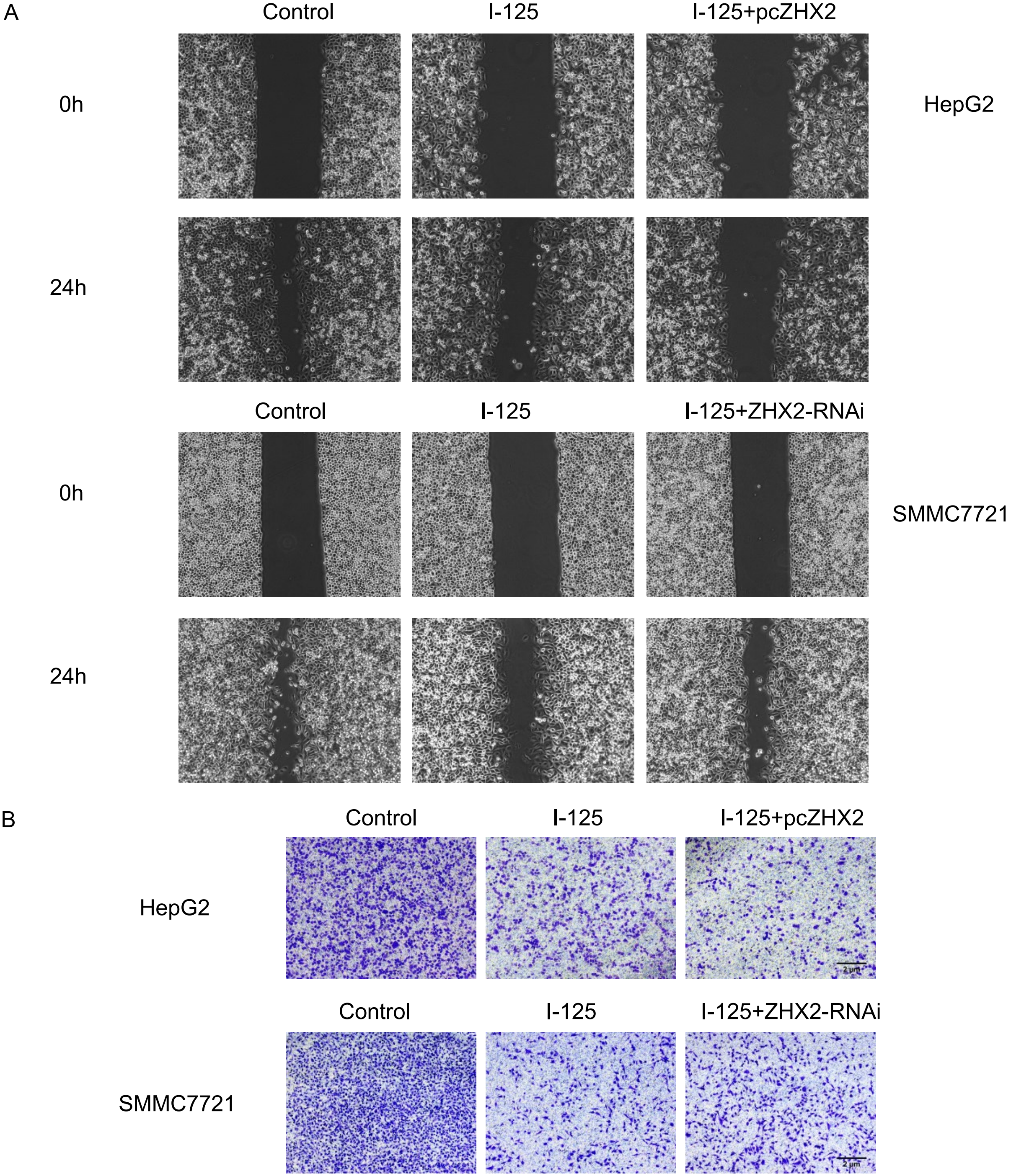
A
Control
I-125
I-125+pcZHX2
0h
24h
HepG2
Control
I-125
I-125+ZHX2-RNAi
0h
24h
SMMC7721
B
Control
I-125
I-125+pcZHX2
HepG2
Control
I-125
I-125+ZHX2-RNAi
SMMC7721

Figure S8

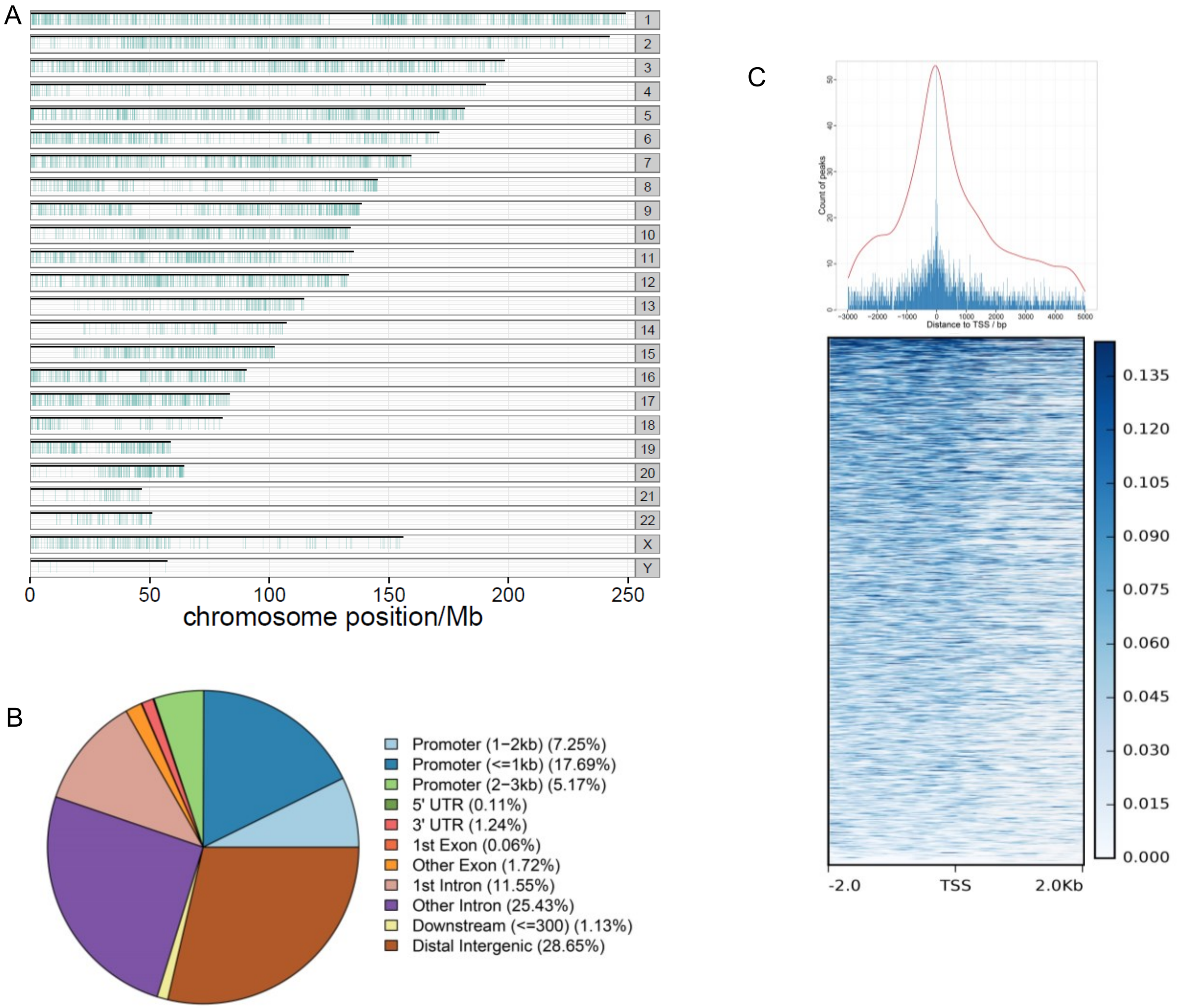

A
1
2
3
4
5
6
7
8
9
10
11
12
13
14
15
16
17
18
19
20
21
22
X
Y
0
50
100
150
200
250
chromosome position/Mb
B
Promoter (1−2kb) (7.25%)
Promoter (<=1kb) (17.69%)
Promoter (2−3kb) (5.17%)
5' UTR (0.11%)
3' UTR (1.24%)
1st Exon (0.06%)
Other Exon (1.72%)
1st Intron (11.55%)
Other Intron (25.43%)
Downstream (<=300) (1.13%)
Distal Intergenic (28.65%)
C
Count of peaks
Distance to TSS / bp
-3000
-2000
-1000
0
1000
2000
3000
4000
5000
0.135
0.120
0.105
0.090
0.075
0.060
0.045
0.030
0.015
0.000
-2.0
TSS
2.0Kb

Figure S9

A

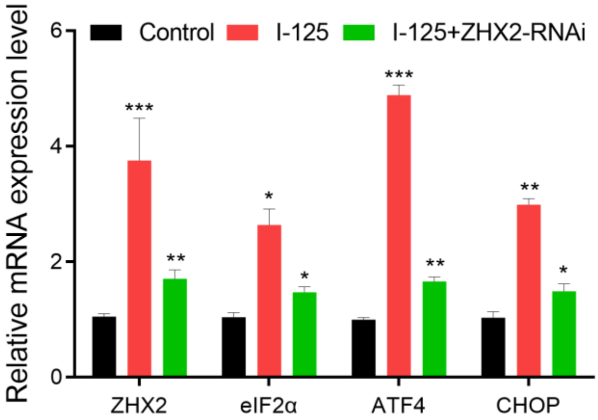

B

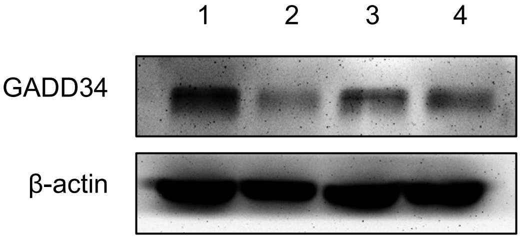

1 Control 2 siRNA-1
3 siRNA-2 4 siRNA-3
