# Supplementary material for: Characterization of circSEC11A as a novel regulator of Iodine-125 radioactive seed-induced anticancer effects in hepatocellular carcinoma via targeting ZHX2/GADD34 axis
Source: Cell Death Discov. 2023 Aug 10;9:294. doi: 10.1038/s41420-023-01593-w (PMC10415397; doi:10.1038/s41420-023-01593-w)
Supplement: Supplementary file 3 — Figure legends for Supplementary figures [file 41420_2023_1593_MOESM3_ESM.docx]

**Figure legends for Supplementary figures**

**Figure S1.** KEGG analysis of circRNA-seq in HepG2 cells treated with I-125.

**Figure S2.** Wound healing assay were performed in HepG2 and SMMC7721 cells treated with I-125 and transfected with sh-NC/sh-circSEC11A or vector/circSEC11A.

**Figure S3. A** Luciferase reporter plasmid of wild type and miR-3529-3p binding site mutant were constructed. **B** Wound healing assay was performed to detect cell metastasis in HepG2 and Huh7 cells treated with I-125 and co-transfected with sh-NC/sh-circSEC11A and miR-3529-3p NC or inhibitor.

**Figure S4.** Wound healing assay was performed to detect cell metastasis in Huh7 cells treated with I-125 and co-transfected with sh-NC/sh-circSEC11A, miR-NC/miR-3529-3p mimics, or pcDNA/pcZHX2.

**Figure S5. A-B** Flow cytometry and TUNEL staining were performed to detect the cell apoptosis in HepG2 and SMMC7721 cells treated with I-125 and transfected with pcZHX2 or ZHX2-RNAi.

**Figure S6. A-B** EdU staining and cell cycle were performed to detect the cell proliferation in HepG2 and SMMC7721 cells treated with I-125 and transfected with pcZHX2 or ZHX2-RNAi.

**Figure S7. A-B** Wound healing and transwell assay were used to verify the cell migration ability in HepG2 and SMMC7721 cells treated with I-125 and transfected with pcZHX2 or ZHX2-RNAi.

**Figure S8. A-B** Reads of ChIP-seq in SMMC7721 cells transfected with ZHX2-RNAi were mapped to the reference genome. **C** The binding sites for ZHX2 were analyzed.

**Figure S9. A** The qPCR was performed to detect the expression level of ZHX2, GADD34, eIF2α, ATF4, and CHOP in SMMC7721 cells transfected with NT-RNAi or ZHX2-RNAi and I-125. **B** The western blot for detecting the expression level of GADD34 in SMMC7721 cells transfected with siRNA 1-3.
