# Supplementary material for: Characterization of circSEC11A as a novel regulator of Iodine-125 radioactive seed-induced anticancer effects in hepatocellular carcinoma via targeting ZHX2/GADD34 axis
Source: Cell Death Discov. 2023 Aug 10;9:294. doi: 10.1038/s41420-023-01593-w (PMC10415397; doi:10.1038/s41420-023-01593-w)
Supplement: Supplementary file 4 — Supplementary Table [file 41420_2023_1593_MOESM4_ESM.pdf]

Table S1. The primer and relative sequence.

cirSEC11A	forward primer AAGGAGATAATAATGCGGTTGATGA<br>reverse primer GGACTTGATAATAGAGCCCCTGG
miR-3529-3p	forward primer CCATCCTACCAACAACAAAATCACT<br>reverse primer TATCCTTCTTCACGACTCCTTCAC
ZHX2	forward primer: 5'-TCGAAACCACCAACCATGTCG-3'<br>reverse primer: 5'-ACCGAGATCCCAGAATCACTG-3'
GAPDH	forward primer GCACCGTCAAGGCTGAGAAC<br>reverse primer TGGTGAAGACGCCAGTGGA
SEC11A	forward primer ACGAGTGGGAGAAATTGTTGT<br>reverse primer CTCTAGCCAATGTTGTCCTTGT
GADD34	forward primer: 5'-ATGATGGCATGTATGGTGAGC-3'<br>reverse primer: 5'-AACCTTGCAGTGTCCTTATCAG-3'
eIF2a	forward primer: 5'-CCGCT CTTGACAGTCCGAG-3'<br>reverse primer: 5'-GCAGTA GTCCCTTGTTAGTGACA-3'
ATF4	forward primer: 5'-ATGACCGAAATGAGCTTCCTG-3'<br>reverse primer: 5'-GCTGGAGAACCCATGAGGT-3'
CHOP	forward primer: 5'-GGAAACAGAGTGGTCATTCCC-3'<br>reverse primer: 5'-CTGCTTGAGCCGTTCATTCTC-3'
U6	forward primer CAGCACATATACTAAAATTGGAACG<br>reverse primer ACGAATTTGCGTGTCATCC
ZHX2-siRNA	ccGTAGCAAGGAAAGCAACAA
pcZHX2	TACCGGACTCAGATCTCGAGCGCCACCATGGCTAGCAAACGAAAATCTACAAC
miR-3529-3p inhibitor	5'-UGGAAGACUAGUGAUUUUGUUGUU-3'
hsa-miR-3529-3p mimics	5'-AACAACAAAAUCACUAGUCUUCCA-3'<br>5'-GAAGACUAGUGAUUUUGUUGUUUU-3'
cirSEC11A-siRNA1	sense GAGCCAGGGGCUCUAUUAUTT<br>antisense AUAAUAGAGCCCCUGGCUCTT
cirSEC11A-siRNA2	sense GAGAGCCAGGGGCUCUAUUTT<br>antisense AAUAGAGCCCCUGGCUCUCTT
cirSEC11A-siRNA3	sense GCCAGGGGCUCUAUUAUCATT<br>antisense UGAUAAUAGAGCCCCUGGCTT
circSEC11A pcDNA	GAATTCTGAAATATGCTATCTTACAGCTCTATTATCAAGTCCTAA<br>ATTTTGGAATGATTGTCTCATCGGCACTAATGATCTGGAAGGGG<br>TTAATGGTAATAACTGGAAGTGAAAGTCCGATTGTAGTGGTGCT<br>CAGTGGCAGCATGGAACCTGCATTTCATAGAGGAGATCTTCTCT<br>TTCTAACAAATCGAGTTGAAGATCCCATACGAGTGGGAGAAATT<br>GTTGTTTTTAGGATAGAAGGAAGAGAGATTCCTATAGTTCACCG

	AGTCTTGAAGATTCATGAAAAGCAAAATGGGCATATCAAGTTTT TGACCAAAGGAGATAATAATGCGGTTGATGACCGAGGCCTCTAT AAACAAGGACAACATTGGCTAGAGAAAAAAGATGTTGTGGGGA GAGCCAGGGGGTGAATATATTTTTTCTTGAGGATCC
FISH	
hsa_circ_0004770 probe	TGAAGAAGGTCCTAATGGGGATGAT
hsa-mir-3529-3p probe	TGGAAGACTAGTGATTTTGTTGTT
RNA pull-down	
hsa_circ_0000647 Probe	5'Biotin-AGGACTTGATAATAGAGCCCCTGGCTCTCCC
NC Probe	5'Biotin-GGGAGAGCCAGGGGCTCTATTATCAAGTCCT
